# Heritable, Prenatal, and Environmental Pathways of Parental Stress: Differential Influences on Children’s Effortful Control and Cortisol Regulation

**DOI:** 10.1371/journal.pone.0355546

**Published:** 2026-08-11

**Authors:** Rebecca E. F. Gordon, Elizabeth J. S. Bates, Jody M. Ganiban, Jenae M. Neiderhiser, Misaki N. Natsuaki, Daniel S. Shaw, Leslie D. Leve

**Affiliations:** 1 Department of Counseling Psychology and Human Services, University of Oregon, Eugene, Oregon, United States of America; 2 Center for Research and Reform in Education, Johns Hopkins University, Baltimore, Maryland, United States of America; 3 Prevention Science Institute, University of Oregon, Eugene, Oregon, United States of America; 4 Department of Psychological and Brain Sciences, George Washington University, Washington, District of Columbia, United States of America; 5 Department of Psychology, Pennsylvania State University, University Park, Pennsylvania, United States of America; 6 Department of Psychology, University of California, Riverside, California, United States of America; 7 Department of Psychology, University of Pittsburgh, Pittsburgh, Pennsylvania, United States of America; Leiden University: Universiteit Leiden, NETHERLANDS, KINGDOM OF THE

## Abstract

Parental stress can have lasting consequences on children’s stress sensitivity and self-regulation capacities. However, it remains unclear the extent to which these effects reflect birth parent influences (genetic and/or prenatal), environmental rearing experiences, and/or interactions between them. Parent–offspring adoption designs offer a powerful framework for disentangling these mechanisms by separating birth parent influences from post-natal environmental pathways when the adoptions occur around the time of birth. Although stress experiences of birth parents and adoptive parents have each been associated with child developmental outcomes, the relationship between them remains unexplored. The present study leverages a parent-offspring adoption approach to examine the associations of birth mother life stress with adolescents’ effortful control (EC) via child diurnal cortisol slopes (a marker of hypothalamic–pituitary–adrenal [HPA] axis functioning) and whether adoptive mothers’ or fathers’ experience of stressful life events moderate this relationship. Participants were drawn from families participating in the longitudinal Early Growth and Development Study. The analytic sample included 561 adopted children, and their birth and adoptive parents. Structural equation modeling revealed that birth mother life stress was linked to poorer adoptee EC in adolescence; neither adoptive mothers’ nor fathers’ experiences of stressful life events predicted adoptee EC. Among adoptive parents, only fathers’ experiences of stressful life events predicted child diurnal cortisol slopes, such that greater adoptive fathers’ stress was linked to flatter adoptee wake-to-bedtime cortisol slopes. However, the hypothesized mediation and moderation effects were not supported. These findings show how the birth parent influences (genetic and/or prenatal) and rearing dimensions of parental stress have differential impacts on the child’s neurobiological functioning and development, where birth mother life stress predicts adolescent self-regulation, and adoptive fathers’ stress predicts child stress sensitivity.

## Introduction

Stress is a pervasive harmful influence that can hinder many aspects of child development. Exposure to chronic or severe stress exposure during early childhood can shape the lifelong trajectories of health outcomes in children, and the stress experienced by parents can have significant indirect effects [1]. Stress may influence offspring development through birth parent prenatal influences (including hormonal and potential epigenetic mechanisms) [2], as well as through rearing environments, including the prenatal environment [1] and the broader postnatal rearing environment [3]. Moreover, heritable factors from birth parents, including stress vulnerability and behavioral tendencies, can influence offspring’s likelihood of experiencing stress or adverse stressful life events, creating intergenerational continuity in stress exposure [1]. Therefore, child development can be impacted directly by cues derived from parental stress in the prenatal environment, as well as by their own lived stressful experiences influenced by the parents’ genetics. Accordingly, the impact of stress on child development transcends generations and warrants investigation within an intergenerational framework.

The direct and indirect effects of parental stress can disrupt children’s physiological stress regulation and undermine the development of key self-regulatory skills, particularly effortful control (EC) [3–5]. EC, the capacity to deliberately shift and focus attention, inhibit dominant responses, and regulate behavior and emotion, is shaped by both biological systems and rearing experiences. One pathway linking stress exposure and self-regulation is hypothalamic–pituitary–adrenal (HPA) axis functioning, often indexed by diurnal cortisol rhythms [6]. However, little is known about the mechanisms connecting parental stress, children’s HPA-axis functioning, and children’s self-regulation.

It is also unclear whether the relationships between parental stress and a child’s development reflect birth parent influences via genetic and prenatal pathways, postnatal environmental rearing experiences, or interactions between the two [1]. Birth parent influences include heritable genetic liability to adverse life experiences (stress risk factors) and genetic sensitivity to stress, as well as prenatal factors stemming from parental lived stress (epigenetics, hormones, or other intrauterine developmental cues) transmitted from birth parents. Parent–offspring adoption designs in which children are adopted around the time of birth offer a powerful framework for disentangling these mechanisms by separating heritable or prenatal pathways from postnatal environmental pathways. Examining how stress from both birth and adoptive parents relates to children’s HPA axis functioning and EC may clarify the distinct contributions of birth parent and rearing parent stress to child developmental outcomes.

### The role of early rearing parent-child relationships

Rearing parent experiences of stressful life events, such as financial hardship, serious illness, or marital instability [7], can reduce the quality of caregiving they provide and shape their children’s developing EC and stress physiology [3]. These stressors may disrupt the neural processing circuitry responsible for children’s emotion regulation and their body’s stress response system [4,5], reduce rearing parents’ emotional availability, and interfere with the formation of secure attachment bonds, in turn, impairing children’s self-regulation skills [8].

The impact of rearing parent stress on child outcomes may differ between mothers and fathers. For example, prior research suggests that paternal exposure to stress may be more strongly associated with child temperament [9], whereas maternal parenting stress is often more closely linked to early childhood behavioral problems [10]. Further, theoretical models of parenting also suggest that fathers and mothers may differ in how stress and personal resources affect their caregiving, thereby shaping different developmental outcomes for their children [11]. Therefore, evidence suggests that maternal and paternal supportive environments differentially shape children’s functional responses to stress. Taken together, examining maternal and paternal experiences of stress separately may be critical for capturing potential differential or additive influences on children’s HPA axis functioning and self-regulation.

### Birth parent influences affecting child stress sensitivity: heritable and prenatal pathways

In addition to postnatal rearing influences, children’s stress sensitivity may reflect inherited and/or prenatal influences. In regard to prenatal influences, maternal stress exposure during pregnancy can shape fetal HPA axis development and heighten later vulnerability to stress [12]. For instance, stress-responsive hormones such as placental corticotropin-releasing hormone and diurnal cortisol can alter fetal programming and elevate risk for stress-related problems across development [13]. These early influences may predispose children to heightened reactivity or dampened regulation but may depend on factors in the rearing environment. Influences on stress sensitivity, whether stemming from parental lived stress or from an intrinsic genetic predisposition, may also be transmitted to offspring either through gametes or via the uterine environment during pregnancy, shaping offspring stress sensitivity [1,2].

Building on these prenatal and genetic influences from birth parents, differential susceptibility theory [3] suggests that some children are biologically predisposed to be more sensitive to their environments—whether for better or worse. According to this framework, the developmental impact of prenatal stress depends on the quality of the postnatal environment [14]. Longitudinal evidence suggests that children exposed to prenatal stress who experience positive or nurturing environments often show more adaptive developmental outcomes compared to those in less supportive contexts [14]. From this perspective, prenatal stress does not uniformly predict maladaptive outcomes. Instead, it may heighten sensitivity to both adverse and supportive postnatal contexts. Thus, prenatal stress, combined with a supportive postnatal environment, may increase developmental plasticity rather than confer risk alone.

### The role of effortful control in adolescence

Individual differences in the functioning of stress response systems are often linked to self-regulation capacities such as EC [15]. EC is a component of self-regulation characterized as both a temperamental trait and a regulatory mechanism that develops throughout childhood and is shaped by both heritable and environmental factors [16]. EC is conceptually related to executive function (EF). While both constructs involve cognitive control processes, EC is typically conceptualized within temperament frameworks as a trait-like capacity for voluntary control of emotion, attention and behavior, whereas EF refers to a set of specific cognitive processes that underlie voluntary self-regulation (e.g., working memory, inhibition, cognitive flexibility) [16]. EC and EF share substantial overlap and likely reflect common underlying neural systems, but EC research emphasizes individual differences in goal-directed self-regulation in everyday contexts, whereas EF research emphasizes cognitive task performance [16].

EC includes a variety of cognitive skills linked to inhibitory control (i.e., ability to inhibit or initiate a dominant motor, vocal, emotional, or cognitive response) and attention regulation (i.e., ability to shift and focus attention deliberately). These capacities develop throughout childhood, with substantial improvements occurring during middle childhood and early adolescence as prefrontal cortical regions mature [17]. By adolescence (ages 10–14), EC capacities are relatively well-established, though they continue to be refined through emerging adulthood [17]. These basic cognitive skills are necessary for the emergence of more complex cognitive abilities and essential for managing distractions, staying focused on tasks, and adapting to changing demands [16]. Further, EC is a key predictor of EF in childhood [18] and adolescent externalizing behaviors [19]. Additionally, lower EC is positively linked to increased risk-taking and impulsivity in adolescence [16] and can exacerbate children’s psychological and emotional maladjustment, especially within a stressful rearing environment [20].

EC has traditionally been considered as a moderating factor in the parent-child stress and development framework. In this sense, EC has been shown to improve children’s long-term stress sensitivity [15], potentially reducing the negative impact of stress exposure on self-regulatory processes [17]. Self-regulation (e.g., attention and inhibitory control), including EC, can buffer the impact of stress exposure, including parental influences (e.g., maternal anxiety, interparental conflict) and a child’s broader ecology (e.g., exposure to violence at school and the neighborhood) on behavior problems. Children with better self-regulation have a lower risk for behavior problems in the context of maternal anxiety and other environmental stressors [21,22]. In this way, EC is usually considered to operate as a mechanism that reduces the impact of stress on behavior and self-regulation.

However, evidence suggests that self-regulation (e.g., EC) itself may be negatively impacted by stress exposure [23], creating a feedback loop in which stress degrades the very regulatory mechanisms that typically buffer its effects. Chronic or early stress exposure may impair EC through repeated activation of the HPA axis which can lead to structural and functional neurobiological damage [24]. Over time, these stress-related neurobiological alterations can negatively impact children’s ability to regulate attention, emotion, and behavior. While this phenomenon is well documented in the context of direct childhood stress exposure, less is known about whether intergenerational stress pathways, stemming from parents’ own experiences of stressful life events, contribute to diminished EC in offspring.

Despite evidence linking EC to both postnatal environmental and heritable or prenatal influences, few studies have disentangled the relative contributions of birth parent-linked vulnerabilities (e.g., genetic and prenatal effects) and postnatal rearing experiences. Adoption designs provide a unique methodological advantage to disentangle the intergenerational effects of stress and stress risk factors. When children are reared apart from their birth parents from around the time of birth, researchers can distinguish the effects of birth parent stress exposure (e.g., via birth mother life stress) and birth parent stress risk factors from rearing environmental stress (e.g., via adoptive parents’ experiences of stressful life events) [1]. For instance, if birth mother life stress is associated with offspring EC or cortisol reactivity, this may reflect genetic risk factors and/or prenatal transmission mechanisms [25]. In contrast, associations with adoptive parents’ experiences of stressful life events reflect rearing environmental influences. Moreover, this design also allows us to evaluate interactions between birth parent-linked vulnerabilities (genetic and/or prenatal) and post-natal rearing environmental influences in predicting child outcomes. It is important to note that in this type of adoption design, associations between birth mother characteristics and offspring outcomes may reflect multiple pathways: (1) genetic transmission of intrinsic vulnerabilities and risk factors, (2) prenatal environmental influences (e.g., maternal stress hormones affecting fetal development), and (3) epigenetic mechanisms that may bridge genetic and environmental processes [14]. In comparison, adoptive parent characteristics can influence children’s postnatal rearing environments. Moreover, because adoptive parents in this study were not genetically related to the child, postnatal environmental influences on children are distinct from heritable and prenatal environmental influences, especially when the adoptive parents and typically assume care of the child within the first few days of the child’s life.

### HPA Axis Functioning, Cortisol Diurnal Variation, and Stress Sensitivity Variability

We have established that children’s EC is a vital component of resilience and development, and it may be impacted not just by heritable, prenatal, and postnatal environmental influences derived from parental stress, but also by interactions between them. However, this framework lacks a clear biological mechanism linking stress exposure to the developmental processes underlying EC. Studies have shown that greater exposure to family adversity, and not simply shared heritable influences, is associated with children’s HPA axis dysfunction [25]. Thus, the HPA axis represents a robust candidate for a mediating factor, as it is both sensitive to parental influences and critical to the development of self-regulation capabilities.

HPA axis functioning is often assessed through physiological markers such as cortisol, a hormone naturally produced by the body that is highly sensitive to stress in the environment. Dysregulated cortisol patterns, such as heightened reactivity, can signal HPA axis dysregulation. For instance, heightened cortisol reactivity has been linked to a range of maladaptive outcomes, including poor executive and cognitive functioning in adolescence [26], increased physiological stress in childhood (i.e., allostasis) [27], higher adiposity among preschool-aged, low-income children [28], and changes in long-term brain structure and functioning [6]. Diurnal cortisol slope, representing the rate of cortisol decline from morning to bedtime, captures individual variability in HPA axis functioning and stress sensitivity [4,29]. Cortisol follows a natural diurnal rhythm, peaking shortly after waking and gradually declining across the day. A flatter slope (i.e., less steep decline) reflects HPA axis dysregulation and has been linked to chronic stress and worse health outcomes [4]. Research on diurnal cortisol slopes and their associations with developmental outcomes has yielded inconsistent findings. While some studies find that flatter slopes (indicating dysregulated HPA axis functioning) predict poorer self-regulation and increased psychopathology risk, others report null associations or context-dependent effects. These inconsistencies may reflect methodological variability (e.g., sampling protocols, age of assessment), individual differences in stress sensitivity, or developmental timing effects. Despite these inconsistencies, diurnal cortisol slope remains one of the most widely used and valid markers of HPA axis functioning in developmental research [27].

Early childhood is an important period for calibrating cortisol rhythms. By preschool age, children typically exhibit a stable diurnal cortisol rhythm [25], making this a critical window for examining early stress physiology. Early stress exposure during this period has been shown to predict HPA axis functioning through adolescence and adulthood [29,30], yet few studies have examined whether cortisol may link heritable stress exposure to adolescent self-regulation and EC. Thus, measuring cortisol slopes at an early age is critical in order to detect the impact of parental risk on child stress sensitivity.

Interestingly, not all children respond to stress in the same way. While some stress-exposed children show a heightened sensitivity to stress (i.e., reflecting biological sensitivity to the environment) [3], others demonstrate a dampened response (i.e., reflecting an adaptation of the stress response as a consequence of down-regulation of stress receptors following chronic stress exposure) [4]. Such variability suggests that stress systems may operate differently for some children or under certain conditions, such as children exposed to more or less prior stress and the quality of their rearing environment.

In addition, the effect of parental stress on child cortisol may differ between mothers and fathers. For instance, father-specific influences on child cortisol reactivity have been identified through parenting behaviors, such as negative or intrusive parenting [31]. Other findings show that greater maternal supportive emotion socialization predicts lower total cortisol output in children, whereas greater paternal supportive emotion socialization predicts higher total cortisol output, indicating potentially distinct pathways by which mothers’ and fathers’ behaviors influence HPA-axis functioning [32]. Together, this evidence highlights the need for research that disentangles maternal and paternal rearing sources of stress when examining children’s HPA-axis functioning and self-regulation. Given the heritable nature of HPA axis functioning and its established links with child EC, it may represent a mediating pathway through which both heritable and environmental stress exposures shape the development of child EC.

### Present study

The current study leveraged a longitudinal parent-offspring adoption design using moderated mediation to examine how heritable factors contribute to adolescent EC via early childhood stress sensitivity, and whether experiences of stressful life events in the rearing environments amplify these effects. This design allows us to separate birth parent influences (measured by birth mother life stress) from rearing influences (adoptive mothers’ and fathers’ experiences of stressful life events) on children’s stress sensitivity and later self-regulation. Specifically, we examined whether birth mother life stress and adoptive parent experiences of stressful life events (mother and father report when children were 27 months old) predict the adoptee’s effortful control in adolescence (age 11), and whether these effects were mediated by child diurnal cortisol slope at age 4.5. We also examined whether adoptive mothers’ or fathers’ experience of stressful life events, assessed via recent negative life events, moderated these pathways.

**Fig 1:**
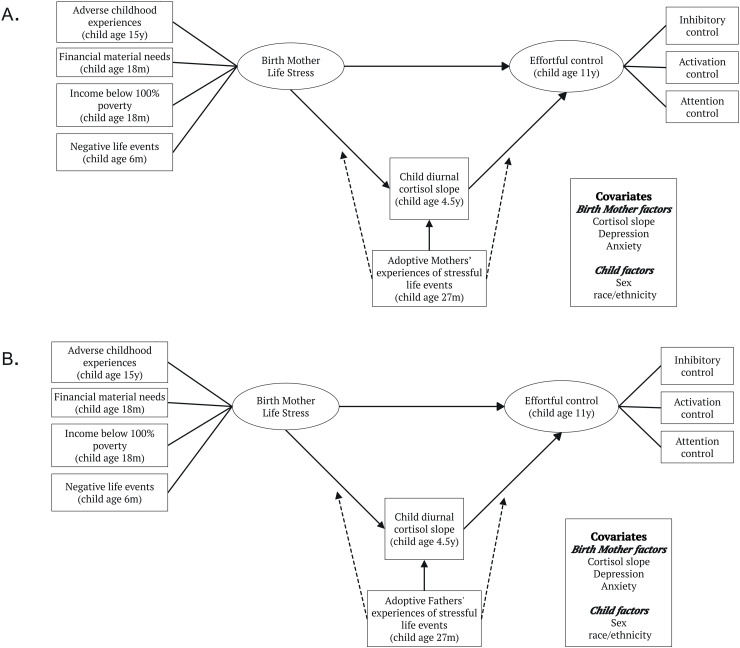
Conceptual figure of the hypothesized pathways from birth mother life stress on adolescent effortful control (EC) mediated by child diurnal cortisol, and moderated by rearing parent experiences of stressful life events. Panel A shows the model with adoptive mothers’ stress as moderator; Panel B shows the model with adoptive fathers’ stress as moderator.

We sought to examine a) whether birth mother life stress would be associated with adolescent EC (11 years old), b) whether HPA axis (i.e., child stress sensitivity) as measured by diurnal cortisol slopes would mediate associations between birth mother life stress and adolescent EC, and, c) whether adoptive parents’ experiences of stressful life events would amplify the pathways in a) and b). Fig 1 illustrates the conceptual framework for the present study. We hypothesized that 1) higher levels of birth mother life stress would predict poorer offspring EC in early adolescence, 2) higher adopted child stress sensitivity, as indexed by child diurnal cortisol slopes at age 4.5, would mediate the relationship between birth mother life stress and offspring EC in early adolescence, 3) higher adoptive parents’ experiences of stressful life events would predict flatter child cortisol slopes at age 4.5, and 4) higher adoptive parents’ experiences of stressful life events (examined separately for mothers and fathers) would amplify the negative effects of child stress sensitivity on EC, such that the mediated association would be stronger when adoptive parent stress is high. For Hypotheses 3 and 4, we expected that maternal stress would more strongly moderate pathways to adolescent EC than adoptive fathers’ stress.

## Methods

### Sample

Participants were drawn from linked birth parents and adoptive families participating in the prospective, longitudinal, Early Growth and Development Study [33]. The present study involved a retrospective analysis of de-identified data collected previously by EGDS investigators. Some authors contributed to the original data collection; however, no identifying information was available to any authors for the current analyses. Recruitment of birth and adoptive families occurred between March 2003 and January 2010. Eligibility criteria included: 1) domestic adoption placement 2) infant placement within three months postpartum, c) infant placement with a nonrelative adoptive family, 3) birth and adoptive parents were able to read or understand English at the eighth-grade level, and 4) the infant had no known major medical conditions such as extreme prematurity or extensive medical surgeries.

### Participants and demographics

The analytic sample included 561 children (42% female, 55% non-Hispanic White, 19% multiethnic/other, 13% Black/African American, and 13% Hispanic or Latino), and their birth and adoptive parents. At study enrollment (infancy, *M* age of placement = 5.58 days, *SD* = 11.32; range = 0–91 days), adoptive families were predominantly non-Hispanic White (over 90%), the median total household income was over $100,000, 2% were single or not married, and the median educational attainment was at least a 4-year college degree for both adoptive mothers and fathers. Adoptive mother’s age averaged 37.4 (*SD* = 5.60) at birth; 92% were non-Hispanic White, 4% were Black/African American, 2% were Hispanic or Latino and 2% were multiethnic/other. Adoptive father’s age averaged 38.3 (*SD* = 5.8) at birth, 90% were non-Hispanic White, 5% were Black/African American, 2% were Hispanic or Latino and 3% were multiethnic/other; 7% (*n* = 41) were same-sex couples. Biological mothers were on average 24.4 years old (*SD* = 6.0 years) at childbirth, and 71% were non-Hispanic White, 13% were Hispanic or Latino, 10% were Black/African American, and 6% were multiethnic/other. At the time of study enrollment, 32% had a total household income of less than $15,000 and 54% had at least a high school degree. In-person assessments with adoptive and birth parents started in infancy, and later assessments were conducted remotely and online. Further information regarding recruitment, consent, procedures, and sample is available elsewhere [33]. The current study used data from the age 4.5- and 11-year-old child assessments, along with birth parent and rearing parent reports collected across early childhood and adolescence. All adult participants (parents/guardians) were provided an information sheet prior to the beginning of an online survey by which data about the child were collected. Consent was implied if the parent or guardian decided to complete the online survey.

This study involved secondary analyses of de-identified data from the Early Growth and Development Study (EGDS), a longitudinal, multisite adoption study. All original data collection procedures were approved by the Institutional Review Boards (IRBs) at participating institutions, including the University of Oregon, Penn State University, and other collaborating sites (approved protocol #08082016.007). Written informed consent was obtained from all participating adults, and written assent was obtained from children when appropriate, at the time of original data collection. The current secondary analyses were deemed exempt from additional IRB review because they involved only deidentified data. All procedures were conducted in accordance with the ethical standards of the institutional and national research committees and with the 1964 Helsinki declaration and its later amendments.

## Measures

### Predictor

*Latent Construct of Birth Mother Life Str*ess. We used a 4-component construct of birth mother life stress that was designed by Leve and colleagues [1] which captures childhood trauma and life stressors that birth mothers experienced before the age of 18, as well as stress that is generally more stable (e.g., income level) but reported within the first 18-months after the adoptee’s placement. The rationale for this construct is that stress experienced by biological mothers, including stress during pregnancy that may have affected fetal development through the intrauterine environment, may predict psychopathology in offspring via genetic (stress vulnerability and risk factors) and prenatal transmission pathways derived from lived stress experiences. This approach helps to elucidate how these mechanisms might carry the effects of maternal stress intergenerationally to offspring. The construct included one variable collected at 3–6 months postpartum (negative life events), two variables collected at 18 months postpartum (financial material needs, household income below the U.S. federal poverty level); and one measure collected approximately 15 years postpartum regarding recalled self-report of ACEs regarding experiences during their own childhood (before age 18). The four components of birth mother life stress are further described below.

Negative life events were assessed using a standard checklist where participants indicated whether they experienced thirty-two stressful events in the past year [34]. Each item was coded as binary (0 = did not occur; 1 = did occur) and summed to produce a negative life events total score with a possible range of 0–32. Inter-item reliability in this sample was acceptable (α = .75).

Financial material needs were assessed using a six-item subscale of the Financial Satisfaction Questionnaire [35] to indicate whether the family had insufficient money to cover material needs (e.g., for housing, clothing, etc.). The scale included six items (e.g., having enough money to afford food) rated on a 5-point Likert scale, where higher scores indicated greater neediness. This scale had acceptable inter-item reliability (α = .87).

Participants self-reported their household income to indicate whether they were living below the U. S. federal poverty level based on household size and composition at the time of data collection, which was binary coded (0 = falls above, 1 = falls below).

Finally, recalled adverse childhood experiences (ACEs) were assessed by the ACEs questionnaire [36], where biological mothers indicated retrospectively whether they experienced ten types of ACEs comprising emotional, physical, and sexual abuse, emotional and physical neglect, and familial dysfunction during their own childhood (before age 18). Responses were binary (0 = did not occur; 1 = did occur) and summed to produce a total ACEs score with a possible range of 0–10. Inter-item reliability for recalled ACEs in this sample was acceptable (α = .84). As done in prior research [1], the four birth mother life stress components were standardized and included in the analyses as a latent construct.

### Mediator

#### Child Cortisol (child age 4.5 years).

Morning and evening saliva samples were collected from the adopted children for three consecutive days and accompanied by a daily diary as part of the age 4.5-year assessment (*M* morning time = 7:36 a.m., *SD* = 42 mins; *M* evening time = 8:12 p.m., *SD* = 53 mins). Adopted children (with the help of adopted parents) were instructed to provide morning saliva samples within 30 minutes after waking (wake time range = 5:00–10:15 a.m.) but before breakfast, and evening saliva samples at bedtime before brushing their teeth (sleep time range = 6:35 p.m.–12:30 a.m.). On average, morning saliva samples were collected 19 minutes after waking (*SD* = 10 mins; range = 0–60 mins) and evening samples were collected, on average, 12 hours, 57 minutes after waking (*SD* = 42 mins; range = 11 hrs, 10 mins –14 hrs, 28 mins). We excluded 7 participants whose morning saliva samples were collected more than 60 minutes after waking to ensure that the analysis focused solely on the diurnal pattern of cortisol, without the influence of the cortisol awakening response [4].

Adoptive parents were trained in sample collection, which involved saturating salivettes before placing them in prelabeled plastic vials. Samples were stored by participants in their refrigerator and then mailed to the primary study site and frozen until all samples for the assessment wave were collected and could be mailed jointly to the analysis laboratory. Then, all samples were sent in bulk from the study site to the University of Trier Laboratory and frozen at −5° F (−20° C) until being used for cortisol immunoassay [37]. Inter-assay coefficients of variance ranged from 3.9–11.7%. Parents recorded the exact time of saliva collection and other information that could affect cortisol measurement, such as illness, medication use, and sleep time, in the daily diary. Samples were assayed in duplicate, and diurnal cortisol slopes (i.e., wake–to–bedtime) were derived by calculating the mean absolute change or rate of change in cortisol from the morning sample to the bedtime sample across the three days of samples [4]. This approach preserves within-person variability and aligns with typical cortisol modeling strategies in young children. Raw Mean (*SD*) morning values (μg/dL) across the three days for each assessment ranged from, respectively,.55 (.33) to.59 (.29). Average evening values ranged from.07 (.15) to.09 (.11).

#### Moderator.

*Rearing parent experience of stressful life events (child age 27 months)* was self-reported independently by both adoptive parents using the Negative Life Events Scale [34] when children were 27 months old. The Negative Life Events scale includes 31 items that assess important stressful life events that the adoptive mother and father may have experienced in the past year, using a dichotomous (yes/no) response option (e.g., “Did you get beaten up, physically attacked, or sexually assaulted?”). Responses were summed and a higher score indicated increased experience of stressful life events. Inter-item reliability in this sample was modest for both adoptive parents (adoptive mothers α = .44; adoptive fathers α = .58) which is typical for life events checklists, as items are not necessarily expected to correlate. Same-sex adoptive couples (*n* = 34; 6% of the sample) were included in the analyses and variables were analyzed based on self-reported parental role (i.e., mother or father). Scores for adoptive mothers’ and fathers’ experiences of stressful life events were assessed in separate models to examine potential differential effects.

### Youth EC Outcome

#### Latent Effortful Control (child age 11 years).

The Effortful Control subscale of the self-reported Early Adolescent Temperament Questionnaire-Revised (EATQ-R) [38] was used to assess three domains of adolescent functioning when children were 11 years old. The three subscales used for this study were activation (eight questions), attention (seven questions), and inhibitory control (eleven questions). The *activation control* subscale is defined as the capacity to perform an action when there is a strong tendency to avoid it, the *attention control* subscale is defined as the capacity to focus attention as well as to shift attention when desired, and the *inhibitory control* subscale is defined as the capacity to plan and to suppress inappropriate responses. Children rated themselves on a 1–5 Likert scale on a series of five items assessing their ability to plan and inhibit inappropriate responses (e.g., “is able to stop him/herself from laughing at inappropriate times”), with higher scores reflecting greater control. Inter-item reliability in this sample was acceptable (inhibitory control: α = .70; attention control: α = .60; activation control: α = .62), with stronger reliability for the overall EC construct (α = .83). Although there was higher internal consistency of the overall score, we modeled EC as a latent construct using the three subscales as indicators. The rationale for this approach is that using a latent variable model captures the shared variance across these distinct yet related domains of regulation while accounting for measurement error, providing a broader representation of EC as opposed to using a single summed score. Further, evidence suggests questionnaire-based assessments often reflect overlapping processes across subdomains, especially attention and inhibition [39]. Thus, constructing a latent factor allowed us to retain subscale-specific information while representing the broader EC construct in a way consistent with our structural equation modeling approach.

### Covariates

Child sex assigned at birth categories (female-assigned and male-assigned) and child race/ethnicity were included as covariates, given the sex differences in EC [40], and that Black and Hispanic adolescents have flatter diurnal cortisol slopes [41]. Child sex assigned at birth was dummy coded into two categories (0 = male-assigned; 1 = female-assigned) and race/ethnicity was dummy coded into four racial-ethnic groups (Black/African American, White, any participants that identified as Latina/o/e/x and/or Hispanic, and/or any other race/ethnicity).

To additionally account for prenatal influences related to their own pregnancy stress experiences, birth mother prenatal anxiety and depressive symptoms were included as covariates. Birth mother prenatal anxiety was retrospectively assessed at enrollment with a subset of items from the Beck Anxiety Inventory (BAI) [42] and birth mother prenatal depression was retrospectively assessed using the Beck Depression Inventory (BDI) [43]. Birth mother diurnal cortisol was also included as a physiological indicator of stress, calculated using the same collection, storage, and assay procedures described for child cortisol. Specifically, birth mother cortisol samples (*n* = 291) were collected at the age 4.5 month post-placement assessment. Diurnal cortisol slopes were derived by calculating the mean absolute change in cortisol from morning to bedtime samples across up to three days of collection.

### Statistical analysis

Structural equation path models (SEM) were estimated in Rstudio (version 4.2.1) using the *lavaan* package for SEM (version 0.6-12) [44,45]. Initial data screening showed non-multivariate normality, and models were estimated with maximum likelihood with robust standard errors (MLR). A confirmatory factor analysis (CFA) in which the latent factors were allowed to covary, fit the obtained data well, such that the parameter estimates were significant for the latent constructs, EC, and birth mother life stress.

First, we fit a mediation model to examine whether child diurnal cortisol slope mediated the association between birth mother life stress and adolescent effortful control (EC). Covariates (child sex, race/ethnicity, birth mother anxiety, depression, cortisol, as well as adoptive parents’ experiences of stressful life events) were included as predictors of both the mediated path (child diurnal cortisol slope) and the direct path to the outcome (adolescent EC) to control for shared variance and reduce confounding.

Next, we fit a dual moderated mediation model to evaluate whether adoptive parent experiences of stressful life events amplified the mediation effects on both the proximal and distal sides of the mediator of the indirect path. Based on prior evidence suggesting potential differential effects by gender [46], we evaluated whether adoptive mothers’ and fathers’ stressful life events effects on child outcomes should be modeled together or separately. Specifically, we evaluated dyadic equivalence to determine whether their effects on child outcomes could be considered statistically equivalent or required distinct estimation. We first examined bivariate correlations between adoptive mothers’ and fathers’ experiences of stressful life events to assess shared variance. To test dyadic equivalence, we conducted chi-square difference tests (χ 2 Δ) on nested models to determine, comparing constrained models (where paths from mothers and fathers were set equal) to unconstrained models (where paths were free to vary). A non-significant chi-square difference test (i.e., *p* = > .05) indicates that the dyadic equivalence is supported and mothers and fathers have statistically indistinguishable effects on each of the tested paths. In contrast, a significant chi-square difference test (i.e., *p* = < .05) indicates that their effects statistically differ for mothers and fathers and should be estimated separately.

For the indirect pathway mediated through child diurnal cortisol slopes, the chi-square difference test was significant (χ²Δ [1] = 3.9, *p* = .04), suggesting differential effects of adoptive mothers’ and fathers’ stress on child cortisol. Accordingly, moderated mediation models were conducted separately for mothers and fathers to assess whether adoptive parent stress amplified the indirect effect of birth mother life stress on adolescent EC. For the moderated mediation models, the proximal and distal indirect effects were tested with bias-corrected bootstrapped (*n* = 1,000), an expectation maximization algorithm to obtain parameter estimates and standard errors, and 95% confidence intervals (CI) for the indices. The same covariate structure was applied across both models for consistency and comparability. Interaction terms were tested to evaluate moderated mediation on both the proximal path (birth mother life stress × adoptive parent stressful life events predicting child cortisol slope) and the distal path (adoptive parent stressful life events × child cortisol slope predicting adolescent EC). Because none of these interaction terms were statistically significant, simple slopes were not probed.

### Missing data

As with most longitudinal studies, some data were missing across time points. Among 561 cases, there were 84 (15%) complete and 477 (85%) partial data cases. Specifically, birth mother life stress data ranged from *n* = 314−538 cases per variable, adolescent EC data ranged from *n* = 387−390, *n* = 283 had child diurnal cortisol data, and adoptive parents’ experiences of stressful life events data ranged from *n* = 494−468 for mothers and fathers, respectively. To test whether data were missing completely at random (MCAR), Little’s MCAR test was used on the sample of participants and indicated that data on variables used in all analyses reported were missing completely at random (*χ*^*2*^(676) = 725.5, *p* = .091), therefore full information maximum likelihood estimation was used to handle the missing data.

## Results

### Confirmatory Factor Analysis for Latent Variables

EC: The CFA showed excellent fit (RMSEA = .00, CFI = 1.0, SRMR = .00), with standardized loadings ranging from.72 to.83. Factor scores from the latent variable were extracted and used in the final model. The CFA measurement models (not shown here) had strong positive relationships for EC: attention control (B = 0.739, *p* < .001) and activation control (B = 0.715, *p* < .001), while inhibitory control served as the reference indicator with a fixed loading of 1.00.

Birth Mother Life Stress: The CFA showed good fit (RMSEA = .02, CFI = .97, SRMR = .02), and revealed moderate factor loadings for the following variables: negative life events (B = 0.470, *p* = .013), income below 100% U.S. federal poverty level (B = 0.506, *p* < .001), and recalled ACEs (B = 0.528, *p* = .023). Financial material needs served as the reference indicator with a fixed loading of 1.00.

### Bivariate correlations

**Fig 2:**
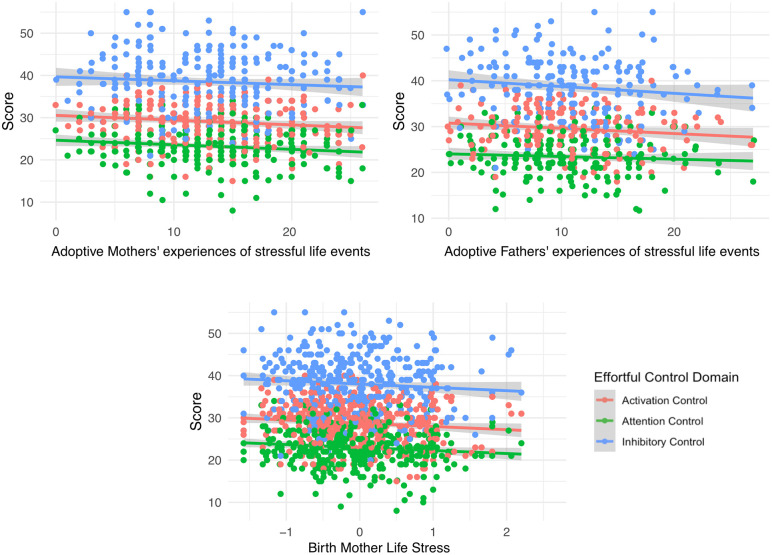
Scatterplots depicting the bivariate associations between stress exposure and adolescent effortful control (EC) domains. Each panel illustrates the bivariate relationship between one source of stress (birth mother life stress, adoptive mother stressful life events, or adoptive father stressful life events) and each EC subscale (activation control, attention control, or inhibitory control) assessed at age 11 years. Lines represent linear regression fits with 95% confidence intervals (shaded regions). Birth mother life stress shows negative associations with activation and attention control, while adoptive parent stress shows minimal associations with EC domains.

Descriptive statistics for all study variables and Pearson’s correlations (*r*) are presented in Table 1. Bivariate associations among study variables were examined using Pearson product-moment correlations for continuous variables. For associations between categorical variables (child sex, race/ethnicity) and continuous variables, point-biserial correlations were computed. Child sex was binary coded (0 = male-assigned; 1 = female-assigned), allowing for point-biserial correlation estimation. Race/ethnicity was dummy coded into four groups; correlations with continuous variables represent associations with the full categorical variable treated as ordinal for descriptive purposes, though these should be interpreted cautiously given the nominal nature of race/ethnicity. The bivariate correlations revealed that higher birth mother life stress was associated with lower scores in two EC domains: attention control (*r* = −.115, *p* = .028) and activation control (*r* = −.140, *p* = .008), but not inhibitory control (*r* = −.082, *p* = .117). While adoptive parents’ experiences of stressful life events were not correlated with any EC domain, those of adoptive fathers specifically were associated with flatter child diurnal cortisol slopes (*r* = .229, *p* = .005). Fig 2 presents scatterplots depicting these bivariate associations between stress exposure (birth mother life stress, adoptive mother and father stressful life events), and the three EC subscales (activation, attention, and inhibitory control). This figure illustrates that birth mother life stress shows modest negative associations with activation and attention control, while adoptive parent stress shows minimal association with any EC domain at the bivariate level.

**Table 1:** Correlation Matrix and Descriptive Statistics of Study Variables.

	1	2	3	4	5	6	7	8	9	10	11	12	13	14
1. Child sex	_													
2. Child race/ethnicity	0.011													
3. Inhibitory control (child age 11y)	0.011	−0.015												
4. Activation control (child age 11y)	0.029	−0.023	**0.525** ^***^											
5. Attention control (child age 11y)	−0.066	0.036	**0.601** ^***^	**0.597** ^***^										
6. Cortisol slope (child age 4.5y)	0.085	**−0.144** ^*^	0.026	−0.008	0.037									
7. BM life stress	−0.066	−0.006	−0.082	**−0.112** ^*^	**−0.113** ^*^	−0.02								
8. BM financial needs	−0.034	−0.028	−0.093	**−0.131** ^*^	−0.089	0.057	**0.717** ^***^							
9. BM below 100% poverty	0	0.01	−0.045	−0.102	−0.085	−0.009	**0.607** ^***^	**0.265** ^***^						
10. BM negative life events	−0.074	0.015	−0.056	−0.059	−0.098	−0.043	**0.682** ^***^	**0.211** ^***^	0.068					
11. BM ACEs	−0.011	0.034	−0.052	−0.094	−0.102	−0.02	**0.655** ^***^	**0.226** ^***^	0.079	**0.205** ^***^				
12. BM Cortisol slope	−0.071	**−0.118** ^*^	0.044	0.045	0.08	0.023	−0.045	−0.064	0.003	−0.056	0.013			
13. Adoptive mothers’ stressful life events	0.019	0.032	−0.008	−0.046	−0.07	**0.144** ^*^	0.018	−0.005	0.073	0.013	0.015	0.044		
14. Adoptive fathers’ stressful life events	0.029	0.016	−0.021	−0.021	−0.03	0.002	0.07	−0.017	**0.148** ^**^	0.06	0.039	−0.016	**0.498** ^***^	_
N	–	–	390	387	387	283	522	494	367	538	314	291	468	494
M	–	–	38.1	28.8	23.0	0.6	7.3	16.6	0.5	6.1	3.7	0.6	1.4	1.5
SD	–	–	6.7	4.8	4.4	0.2	3.2	6.2	0.5	3.8	0.2	0.2	0.8	0.9
Min	–	–	16	15	8	0.3	0.0	6.0	0.0	0.0	0.3	0.3	0.5	0.5
Max	–	–	55	40	34	1.1	21.0	30.0	1.0	19	1.3	1.3	5	5

*Note.* BM = Birth mother; *p < .05; **p < .01; ***p < .001. Correlations are Pearson product-moment correlations for continuous variables. For categorical variables (child sex and race/ethnicity), point-biserial correlations are reported with continuous variables. Child sex was coded as 0 = male-assigned, 1 = female-assigned. Race/ethnicity was dummy coded into four groups (see Methods).

### Model fits

All models were estimated using robust maximum likelihood. The mediation model demonstrated adequate fit on absolute indices (RMSEA = .07, SRMR = .07), but comparative fit was poor (CFI = .77), falling below commonly accepted thresholds (CFI ≥ .90). Similarly, the moderated mediation models demonstrated acceptable RMSEA and SRMR (adoptive mothers: RMSEA = .04, SRMR = .07; adoptive fathers: RMSEA = .04, SRMR = .07), but CFI values remained low (adoptive mothers: CFI = .75; adoptive fathers: CFI = .76). These indices suggest that while the models capture some of the data structure, the overall specification does not fully account for the observed covariances. Consequently, results should be interpreted cautiously, and substantive conclusions drawn from these models are limited. Model outputs and model fits are presented in Tables 2 and 3, respectively.

**Table 2:** Model Outputs.

	Effortful Control (child age 11 years)		
Predictor	b [95% Bootstrap CI]	SE	β
**Main Effects**			
BM Life Stress	−0.46 [−0.793, −0.122]	0.17	−0.41***
Child cortisol slope (child age 4.5y)	1.13 [−1.602, 1.861]	0.88	0.12
Adoptive mothers’ stressful life events (child age 27m)	−0.10 [−0.392, 0.186]	0.15	−0.09
Adoptive fathers’ stressful life events (child age 27m)	−0.12 [−0.285, 0.150]	0.14	−0.10
Child Sex	−0.01 [−0.174, 0.152]	0.08	−0.01
**Indirect, Direct, and Total Effects**			
Indirect effect (a*b)	0.002 [−0.026, 0.030]	0.01	0.002
Direct effect (c’)	−0.46 [−0.793, −0.122]	0.17	−0.41***
Total effect	−0.46 [−0.794, −0.117]	0.17	−0.41***
**Moderated mediation analysis**			
Adoptive mothers’ stressful life events × Cortisol Slope	−0.58 [−1.534, 0.395]	0.48	−0.56
Adoptive fathers’ stressful life events × Cortisol Slope	−0.35 [−1.771, 1.188]	0.73	−0.34

*Note.* BM = Birth mother. *p <.05; **p <.01; ***p <.001. CI = Confidence Interval

**Table 3:** Model Fits.

	*Chi-square Δ*	*Degrees of Freedom*	*p-value*	*Comparative fit index (CFI)*	*Root mean square error of approximation (RMSEA)*	*Akaike information criterion (AIC)*	*Bayesian information criterion (BIC)*
Model 1 (Mediation)	126.511	60	0	0.722	0.069	5114.31	5252.87
Model 2 (Moderated Mediation – Mothers Only)	384.024	197	0	0.747	0.041	27321.2	27762.9
Model 3 (Moderated Mediation – Fathers Only)	363.003	197	0	0.758	0.039	26179	26620.6

### Structural models

#### Direct effects of birth mother life stress on EC.

We first examined the effects of birth mother life stress on adolescent EC. Consistent with Hypothesis 1, birth mother life stress was negatively associated with adolescent EC (β = −0.407, *p =* .007, SE = .171; 95% CI [−0.793, −0.122]). This indicates that adolescents whose birth mothers had higher life stress experienced poorer overall EC across domains, after accounting for model covariates.

#### Mediating role of child diurnal cortisol slope.

To test Hypothesis 2, we assessed child diurnal cortisol slope as a potential mediator of birth mother life stress on adolescent EC. Child diurnal cortisol slope was not significantly associated with adolescent EC (b path) (β = 0.018, SE = 0.883, *p =* .147; 95% CI [−1.602, 1.861]). Further, the indirect effect of birth mother life stress through child diurnal cortisol slope (a*b path) on adolescent EC was not significant (β = 0.002, SE = 0.014, *p =* .892; 95% CI [−0.026, 0.030]). Thus, cortisol slope did not mediate this relationship, providing no support Hypothesis 2. Importantly, the direct effect of birth mother life stress on adolescent EC (c prime path) remained significant after controlling for covariates and the cortisol slope (β = −0.405, *p =* .008, SE = .173; 95% CI [−0.793, −0.122]).

### Direct effects of rearing parents’ experience of stressful life events

**Fig 3:**
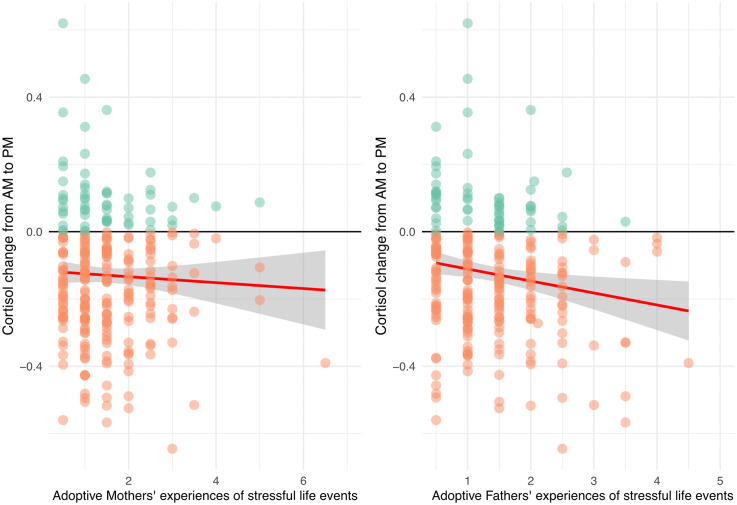
Association between rearing parent experiences of stressful life events (assessed at child age 27 months) and children’s diurnal cortisol slopes (wake-to-bedtime) at age 4.5 years. The figure illustrates that higher adoptive father stressful life events predicted flatter (less negative) cortisol slopes, indicating greater HPA axis dysregulation (β = 0.158, p = .030). Adoptive mother stressful life events were not significantly associated with child cortisol slopes (β = 0.013, p = .761). Lines represent predicted values from the structural equation model; shaded regions represent 95% confidence intervals. More negative slope values indicate steeper (more typical) diurnal decline; values closer to zero indicate flatter (dysregulated) patterns.

To better understand the relational context of Hypothesis 3, we first examined the direct effects of rearing parent experiences of stressful life events on of child diurnal cortisol slopes. Notably, fathers’ experiences of stressful life events emerged as a significant direct predictor (β = 0.158, SE = 0.014, *p =* .030; 95% CI [0.004, 0.060]). As illustrated in Fig 3, higher adoptive fathers’ experiences of stressful life events were associated with flatter (less steep) wake–to–bedtime cortisol slopes in children, indicating greater HPA axis dysregulation. In contrast, adoptive mothers’ experiences of stressful life events showed no significant association with child cortisol slopes (β = 0.013, SE = 0.072, *p =* .761; 95% CI [−0.156, 0.122]), as also shown in Fig 3.

Finally, neither adoptive fathers’ experiences of stressful life events (β = −0.058, SE = 0.088, *p =* .404; 95% CI [−0.235, 0.113]), nor adoptive mothers’ experiences of stressful life events (β = −0.021, SE = 0.072, *p =* .761; 95% CI [−0.704, 8.416]), was significantly associated with adolescent EC, after controlling for model covariates and individual differences in child diurnal cortisol slope.

### Moderation effects of parental experiences of stressful life events

To test Hypothesis 3, we examined the moderating role of adoptive parent stressful experiences on both sides the indirect pathway. On the proximal path to the mediator, the interaction between birth mother life stress and adoptive mother experiences of stressful life events did not significantly predict diurnal cortisol slopes of their children (β = 0.001, SE = 0.091, *p* = .755; 95% CI [−0.059, 0.038]), and neither did the experiences of adoptive fathers (β = −0.002, SE = 0.025, *p* = .988; 95% CI [−0.988, −0.054]). Similarly, on the distal path from the mediator, the interaction between adoptive mothers’ experiences of stressful life events and child diurnal cortisol slope predicting EC paths was not significant (β = −0.054, SE = 0.731, *p =* .631; 95% CI [−1.771, 1.188]), and it was also non-significant in the model considering adoptive fathers (β = −0.084, SE = 0.088, *p =* .404; 95% CI [−1.534, 0.395]).

## Discussion

The present study contributes to a growing body of literature examining how early biological and environmental stressors shape adolescent self-regulation. Specifically, we examined whether child diurnal cortisol slope in early childhood mediated the association between birth mother life stress and adolescent EC, and whether rearing parents’ experiences of stressful life events moderated these pathways. Although hypothesized mediation and moderation effects were not supported, these null results are theoretically informative as they help delineate when and which avenues of parental stress exposure may (or may not) influence children’s biological and behavioral regulation.

### Direct and indirect pathways from birth mother life stress

Our results highlight the importance of birth parent influences in the development of adolescent self-regulation. Specifically, birth mother life stress was associated with lower adolescent EC, even after controlling for adoptive mothers’ and fathers’ experiences of stressful life events and individual child biological factors (i.e., child sex assigned at birth and race/ethnicity). This finding contributes to a broader framework of prenatally transmitted effects on EC by demonstrating that birth parent influences are an important contributor to adolescent development. This pattern suggests that EC may be shaped by multiple pathways, including genetic transmission of regulatory vulnerabilities [46], as well as prenatal environmental influences (e.g., maternal stress hormones affecting fetal HPA axis development and epigenetic factors).

While birth mother life stress was directly associated with adolescent EC, this relationship was not mediated by child diurnal cortisol slope. Notably, the indirect effect was not only non-significant but also negligible in magnitude, suggesting that child diurnal cortisol slopes are unlikely to represent a meaningful biological mechanism linking birth parent stress to adolescent self-regulation in this sample. Although *in utero* exposure to physiological mechanisms for stress (e.g., glucocorticoids, cortisol, inflammatory cytokines, excitatory amino acids, and brain-derived neurotrophic factor) inhibits growth and causes permanent damage to stress-sensitive brain regions such as the HPA and autonomic axes [47], such prenatal programming may not be reflected in early childhood cortisol patterns. Moreover, birth mother cortisol was included as a covariate but was not significantly associated with child cortisol or EC, ruling out the possibility that genetic covariation in HPA axis functioning accounts for the observed association between birth mother stress and child outcomes. These results suggest that if birth mother stress effects operate through biological pathways, they may involve prenatal programming mechanisms not captured by early childhood diurnal cortisol patterns, or genetic transmission of vulnerabilities affecting behavioral rather than physiological stress regulation.

Alternatively, the lack of mediation may also reflect developmental timing differences in assessment (i.e., cortisol was measured at age 4.5 years, adoptive parent experiences of stressful life events at 27 months, and EC age 11 years). Given that HPA-axis regulation and EC may develop and stabilize during different developmental periods [48], this temporal mismatch could partially explain the null mediation effect. Although cortisol levels are usually stable across development [49], patterns of HPA axis functioning can change with age. Cortisol is lowest when youth are 11 years old, while older adolescents have lower morning cortisol and flatter circadian rhythms. Moreover, it is possible that diurnal cortisol slope does not fully capture HPA-axis functioning. Other measures of biological stress sensitivity, such as cortisol awakening response, may be a more sensitive indicator of stress transmission [50]. Future research should incorporate multiple markers of HPA axis functioning across development to better capture stress regulation and its links to heritable and rearing influences on self-regulation.

### Adoptive parent experiences of stressful life events: direct and moderation effects

We hypothesized that rearing parent experiences of stressful life events would moderate associations between birth mother life stress and adolescent EC, mediated by child diurnal cortisol. This hypothesis was not supported by our findings. This contrasts with a recent study conducted by Leve and colleagues [1] which also used the EGDS sample. The authors found that prior adoptive parent stress (measured via recalled ACEs) moderated the effect of birth mother life stress on offspring EC in childhood. In this study, the direct effect of birth mother life stress on adolescent EC was only significant at high levels of adoptive parent stress. This discrepancy between studies may reflect differences in stress measurement (i.e., adverse childhood experiences compared to negative life events) and timing, which may impact our ability to detect its influence on adolescent EC. Early stressful experiences, such as ACEs, which occurred during the adoptive parent’s own childhood, may have a more lasting influence on their psychological functioning and caregiving quality than stressful events experienced in adulthood [51]. Thus, the developmental timing of parental stress exposure may be critical for the development of their child’s self-regulation, even in adoptive families where biological transmission from the rearing parent is not a factor.

Furthermore, the lack of significant moderation effects from adoptive parent experiences may be explained by the nature of regulatory capacities like EC, which may be more directly influenced by immediate, proximal rearing factors, such as specific caregiving qualities like sensitivity [52], than by broader, more distal contextual experience of stressful life events. Thus, EC may be more responsive to current caregiving quality than to rearing parent stressful life events experienced at earlier time periods. For example, prior EGDS research found that the interaction between birth mother emotion dysregulation and adoptive parent laxness (versus structured parenting) predicted emergent EC skills in children [21]. Thus, certain rearing approaches may be required to offset the effects of birth parent stress and heritable stress risk factors on children’s regulatory development. Future research should identify which specific parenting strategies most effectively buffer children from heritable risk and promote the development of EC across contexts of parental stress.

There are several reasons why adoptive parent experiences of stressful life events did not significantly moderated associations between birth mother life stress and adolescent EC. First, there are likely unmeasured rearing factors in the current study that may be more proximal moderators of the birth parent-child EC association within the rearing environment. For example, household factors such as noise or chaos in the home [53], as well as greater positive parenting or less harsh discipline [54] have been shown to influence the effects of early stress exposure on child self-regulation and executive functioning. Additionally, we only examined adoptive parents’ stressful life events, thereby removing the potential for protective environment influences promotive of health and well-being that also shape self-regulation (e.g., warmth) [55], and excluding other forms of stress. Future research should consider a broader range of rearing experiences, including both risk and protective influences, when examining how early stress affects the development of self-regulation.

While the moderation hypothesis was not supported, we did find that adoptive fathers’ experiences of stressful life events directly predicted flatter child diurnal cortisol slopes at age 4.5, whereas adoptive mothers’ stressful life events did not. This finding should be interpreted cautiously for several reasons. First, the effect emerged from separate models for mothers and fathers rather than a direct statistical test comparing adoptive mothers’ versus fathers’ effects within a single model. Second, given the number of pathways tested and the modest effect size (β = 0.158), we cannot entirely rule out Type I error. Third, the overall model fit indices suggest the models do not fully capture the data structure, limiting confidence in specific parameter estimates.

Nevertheless, this finding demonstrates some consistency across analytic approaches and aligns with emerging evidence of father-specific influences on child stress physiology. At the bivariate level, adoptive father stressful life events were significantly correlated with child cortisol slopes, and this association remained significant after controlling for multiple covariates in the structural model. This pattern is consistent with prior research showing that father-specific parenting behaviors, such as negative or intrusive parenting, uniquely predict child cortisol reactivity [31], and that paternal and maternal emotion socialization differentially influence children’s HPA axis functioning [32]. Several explanations warrant consideration. One possible explanation is that differences in caregiving roles, time spent in parenting, or the types of stressors experienced by fathers versus mothers may differentially shape children’s physiological stress responses. It is also important to note that this finding contrasts with the larger body of literature emphasizing maternal influences on child stress physiology, particularly in early childhood when mothers typically provide more direct caregiving. This unexpected finding underscores the need for replication in independent samples and more direct comparisons of maternal versus paternal stress effects using models that simultaneously estimate both parent effects. Future studies should assess whether paternal stress influences children’s biological stress regulation through pathways that differ from maternal influences.

Although some hypothesized pathways were not supported, these findings have implications for preventive intervention and policy, indicating that efforts to improve child self-regulation and resilience should account for both heritable and rearing factors. Our results show how the impacts of parental stress may vary depending on the source of parental stress (e.g., whether it occurs in the context of the child rearing environment versus prenatally). Specifically, birth parent stress impacted EC, while rearing parent stress impacted child cortisol slopes. While cortisol slopes did not predict EC, a dysregulated HPA axis can still have manifold impacts on the health and development of a child. Given that adoptive fathers’ experiences of stressful life events emerged as a significant predictor of children’s diurnal cortisol slopes, interventions that specifically support paternal mental health and stress management may be especially beneficial for promoting healthy biological regulation in children.

Our results show that the impact of stress exposure is carried beyond a single generation, whether it is via prenatal, biological, or rearing transmission pathways. Therefore, it is critical to utilize two-generation intervention approaches that: (1) bolster family strengths, and (2) address parental trauma, stress histories (e.g., parent mental health, ACEs, family economic stability), and specific needs [56]. Given the importance of preventing stress exposure before it reaches children through their parents, it is essential to promote the accessibility of mental health support for parents before stress manifests in parenting practices to prevent deleterious child outcomes.

### Limitations

This study has several notable strengths, including the robust methodology. In particular, its longitudinal adoption research design allowed us to disentangle birth parent influences from rearing parent experiences of stressful life events, which is often challenging in traditional family studies. However, several limitations should be acknowledged. First, the cortisol assessment was conducted once, at child age 4.5 years, and was based on only two samples per day (30 minutes after waking and at bedtime; across 3 days). While the diurnal cortisol patterns are considered to be stable in children and adolescents [57], this limited sampling may not fully capture variability in diurnal cortisol rhythms. Cortisol levels naturally fluctuate throughout the day, with multiple peaks influenced by factors such as stress, activity, and sleep patterns. More frequent sampling, including measurements at multiple points across the day (e.g., midday or afternoon), would provide a richer and more accurate representation of HPA axis functioning. Furthermore, the limited number of collection days may reduce reliability, as day-to-day cortisol secretion can vary substantially. Future research should consider more intensive and repeated cortisol sampling to better capture the temporal patterns and variability of HPA axis activity. Second, the sample primarily consisted of high-SES, two-parent adoptive families with limited racial and economic diversity. Specifically, 55% of the children were non-Hispanic White, and 91% of the adoptive parents were non-Hispanic White. The relatively low range of family adversity and variability in child executive functioning may restrict the generalizability of the findings to more diverse or at-risk populations. Third, the structural equation models used to test mediation and moderation showed only moderate fit. While overall model fit indices were acceptable, some indices suggested only moderate fit, indicating that the models may not fully capture the complexity of the relationships among birth mother life stress, adoptive parent stress, child cortisol, and adolescent EC. This limitation suggests that the null findings for certain pathways should be interpreted with caution, as alternative model specifications or additional variables could reveal different associations. Future research may benefit from larger samples and more comprehensive modeling approaches to better evaluate these pathways. Many of the future research directions we propose require measures that were not available at the relevant developmental time points in this study (e.g., repeated cortisol assessments into adolescence). Fourth, EC was assessed via adolescent self-report, which could introduce bias as questionnaires typically assess trait-relevant, emotionally salient behaviors and may not account for all aspects of the conceptualization of the EC construct [46]. Further, it may only capture executive attention, rather than cognitive control or higher-level executive functioning [16] which limits its definition of potential targets for interventions. This may limit its applicability for interventions aimed at improving adolescent self-regulation, as different EC domains may respond differently to environmental supports. Fourth, this study was not preregistered, which limits our ability to distinguish confirmatory from exploratory findings. The unexpected finding that adoptive fathers’ experiences of stressful life events, but not mothers’, predicted child cortisol slopes emerged from theory-driven analyses. While we used a formal test of dyadic equivalence (chi-square difference test) to justify examining maternal and paternal effects separately, and the father-specific association was significant at both bivariate and multivariate levels, the possibility of Type I error cannot be ruled out given the number of pathways tested. This finding should be considered preliminary and interpreted cautiously pending replication in independent samples.

## Conclusion

In summary, the present study provides evidence that stress from one’s biological parent predicts lower adolescent EC, when the child is not raised with the biological parent, suggesting that birth parent influences operate through genetic and/or prenatal pathways. Although individual differences in HPA axis regulation did not mediate these associations, adoptive fathers’ experiences of stressful life events emerged as a significant predictor of child stress sensitivity. These findings show how the biological, prenatal, and postnatal environmental dimensions of parental stress have differential impacts on the child’s neurobiological functioning and development, where birth parent stress impacts self-regulation, while rearing parent stress impacts stress sensitivity. Recognizing how birth parent vulnerabilities and daily rearing experiences shape child development can help inform the development of more precise, family-centered interventions. Future research should continue to investigate how maternal and paternal experiences of stressful life events differentially influence developmental outcomes and identify the rearing processes that may buffer or exacerbate the impact of birth parent stress on children’s regulatory capacities.
