# Supplementary material for: Heritable, Prenatal, and Environmental Pathways of Parental Stress: Differential Influences on Children’s Effortful Control and Cortisol Regulation
Source: PLoS One. 2026 Aug 11;21(8):e0355546. doi: 10.1371/journal.pone.0355546 (PMC13460627; doi:10.1371/journal.pone.0355546)
Supplement: Supplementary file 1 [file pone.0355546.s002.docx]

May 4, 2026

Ralph C. A. Rippe, Ph.D.

Academic Editor

PLOS One

Dear Dr. Rippe,

Thank you for the opportunity to revise and resubmit our manuscript titled, *Heritable, Prenatal, and Environmental Pathways of Parental Stress: Differential Influences on Children’s Effortful Control and Cortisol Regulation*. We appreciate the thorough review and feedback we received. We have done our best to address all the reviewers’ concerns and believe the resulting manuscript is significantly strengthened. Our responses to reviewer comments are detailed below.

Sincerely,

Rebecca Gordon

Corresponding author

| Comments | Responses |
| --- | --- |
| Journal Requirements: Please ensure that your manuscript meets PLOS ONE's style requirements, including those for file naming. | We have reviewed and ensured that all file naming and formatting complies with PLOS ONE style requirements. |
| Thank you for stating the following financial disclosure: [Data collection for this study were supported by grant R56 HD042608 and R01 HD042608 from the Eunice Kennedy Shriver National Institute of Child Health & Human Development, the National Institute on Drug Abuse, and OBSSR, NIH, U.S. PHS (PI: Leslie Leve, PhD); by grant R01 DA035062 from the National Institute on Drug Abuse (PI: Leslie Leve, PhD) and grant R01 DA020585 from the National Institute on Drug Abuse, the National Institute of Mental Health, and OBSSR, NIH, U.S. PHS (PI: Jenae Neiderhiser, PhD); and by the Environmental influences on Child Health Outcomes program, Office of The Director, National Institutes of Health, under Award Number UH3OD023389 (PIs: Leslie Leve, PhD; Jenae Neiderhiser, PhD; Jody Ganiban, PhD).].  Please state what role the funders took in the study. If the funders had no role, please state: ""The funders had no role in study design, data collection and analysis, decision to publish, or preparation of the manuscript.""  If this statement is not correct you must amend it as needed.  Please include this amended Role of Funder statement in your cover letter; we will change the online submission form on your behalf. | We have added the following statement to the cover letter and will update the online submission form accordingly: "The funders had no role in study design, data collection and analysis, decision to publish, or preparation of the manuscript." |
| In the online submission form you indicate that your data is not available for proprietary reasons and have provided a contact point for accessing this data. Please note that your current contact point is a co-author on this manuscript. According to our Data Policy, the contact point must not be an author on the manuscript and must be an institutional contact, ideally not an individual. Please revise your data statement to a non-author institutional point of contact, such as a data access or ethics committee, and send this to us via return email. Please also include contact information for the third party organization, and please include the full citation of where the data can be found. | We have modified this in order to post a public dataset. Specifically, the de-identified dataset and analysis code are now publicly available via OSF (<https://osf.io/usw93>). |
| When completing the data availability statement of the submission form, you indicated that you will make your data available on acceptance. We strongly recommend all authors decide on a data sharing plan before acceptance, as the process can be lengthy and hold up publication timelines. Please note that, though access restrictions are acceptable now, your entire data will need to be made freely accessible if your manuscript is accepted for publication. This policy applies to all data except where public deposition would breach compliance with the protocol approved by your research ethics board. If you are unable to adhere to our open data policy, please kindly revise your statement to explain your reasoning and we will seek the editor's input on an exemption. Please be assured that, once you have provided your new statement, the assessment of your exemption will not hold up the peer review process. | We confirm our commitment to PLOS ONE's open data policy. As stated above, the de-identified dataset and analysis code are now publicly available via OSF (https://osf.io/usw93). We have consulted with our IRB and confirmed that public sharing of de-identified data is consistent with our approved protocol (University of Oregon IRB #08082016.007) and participant consent procedures. |
| Please include your full ethics statement in the ‘Methods’ section of your manuscript file. In your statement, please include the full name of the IRB or ethics committee who approved or waived your study, as well as whether or not you obtained informed written or verbal consent. If consent was waived for your study, please include this information in your statement as well. | The ethics statement is now included in the Methods section and specifies the approving IRB and the consent procedure (Lines 289-298) |
| **Reviewer #1:** This manuscript is well written and the study has many strengths including a large sample size and use of a longitudinal behavioral genetics study design (parent-offspring adoption design). | We thank Reviewer 1 for the constructive feedback and specific suggestions for improving clarity and comprehensiveness. |
| The purpose of including adoptive mothers/fathers report of stressful life experiences should be clarified in the manuscript. This construct was measured with one questionnaire whereas other main variables in the model were measured using latent variables with multiple indicators. This approach seems to detract from the conclusions that can be made from analyses of parental stressful life events, especially when compared to the more robust measurement approach used for the other variables. This is also problematic as part of the aims of the study center on genetic vs. environmental influences. | We appreciate the reviewer’s point and have clarified the purpose of adoptive parent stressful life experiences in the Introduction (Lines 141-147; 162-164; 228-230). This variable is critical to disentangle the heritable/prenatal (birth parent) effects from the postnatal rearing environmental (adoptive parent) stress effects. We specifically selected discrete stressful life events as our environmental stress indicator because life events represent objectively verifiable environmental exposures that are conceptually distinct from parenting behaviors, parent-child relationship quality, or heritable child characteristics. In order to characterize variation in adoptive parent stressful life experiences, we were able to capitalize on the available measures of adoptive parent stressful life experiences in EGDS during the relevant developmental window (27 months). While multi-indicator latent constructs may provide more robust measurement, modeling this variable as a single indicator was the appropriate approach given the available data. |
| The introduction should differentiate between positive and toxic stress such as manageable vs. overwhelming levels of stress rather than defining all stress as harmful. | We appreciate the reviewer's point. Our Introduction references differential susceptibility theory, which does acknowledge that some children may benefit from positive environments. However, the present study focuses specifically on adverse stress exposure rather than examining the full continuum of environmental quality. Our measures (ACEs, negative life events, material hardship) are validated risk indices designed to capture experiences such as abuse, neglect, and financial instability that have been consistently linked to poor developmental outcomes. These are not measures of normative challenges or positive experiences. While differential susceptibility theory suggests that prenatal stress may heighten sensitivity to both adverse and supportive contexts, testing the "for better" hypothesis would require measures of positive caregiving experiences (e.g., warmth, sensitivity, enrichment), which were not the focus of the present analyses. To clarify our scope, we revised the opening paragraph to specify "chronic or severe stress exposure" (Line 31) |
| It would be helpful to explain the focus on effortful control earlier in the manuscript. | We appreciate this feedback. Our Introduction includes a dedicated section titled "The Role of Effortful Control in Adolescence" that provides a comprehensive definition of EC, its components (inhibitory control and attention regulation), and its theoretical importance before presenting our study hypotheses. This organizational structure allows us to first establish the broader conceptual framework (stress transmission pathways, HPA axis functioning, and adoption designs for disentangling heritable from environmental influences) before focusing on the specific self-regulation outcome (EC) being examined. We believe this structure provides readers with the necessary theoretical context to understand why EC is an appropriate outcome for examining the effects of intergenerational stress transmission. |
| In addition, in multiple places, it seems as though studying adolescent mental health (rather than effortful control) could be more aligned with the authors’ interests and hypotheses. | We appreciate the reviewer's perspective. However, effortful control (EC) was selected as the primary outcome based on strong theoretical and empirical foundations. While mental health outcomes are certainly important, EC represents a more proximal and mechanistic target that can inform our understanding of how parental stress shapes children’s self-regulatory capacities. We have clarified the rationale for focusing on EC in the Introduction (Lines 39-42). |
| On Line 90 of the introduction, it would be helpful to clarify what is meant by “…which peak during adolescence.” It would help to explain more about the development of effortful control before, during, and after adolescence. | We have added a developmental trajectory section to clarify EC development across the lifespan (Lines 108-112). |
| In that same section, the difference between effortful control and executive function should be explained in more detail. The two constructs are overlapping, and some argue that they are different terms for the same underlying construct. | We agree and have expanded the conceptual framing to distinguish EC and executive function while acknowledging their substantial overlap (Lines 98-105). |
| It is important to include in the introduction that studies of salivary cortisol metrics such as diurnal cortisol slope have yielded inconsistent results. | Excellent point. We have added acknowledgment of inconsistent cortisol findings in the HPA Axis section (Lines 191-198). |
| Is there data on effortful control prior to adolescence? Including those data could strengthen the model. | We appreciate this suggestion. The current study was designed to examine adolescent EC as the outcome of interest, specifically focusing on how early stress exposures (birth mother life stress and adoptive parent stressful life events during toddlerhood) predict self-regulatory capacity in adolescence. We focused on age 11 because EC skills, including inhibitory control and attention regulation, peak during adolescence (as mentioned in the Introduction), making this developmental period ideal for examining the cumulative effects of early stress exposure on self-regulatory outcomes. Examining developmental trajectories of EC across childhood would be a valuable direction for future research but is beyond the scope of the current study's research questions, which focus on adolescent regulatory outcomes as a function of early heritable and environmental stress exposure. |
| What is shown in Figure 4 is not clear. It would help to clarify the reason for including this figure. | We agree that Figure 4 (loop plot of non-significant moderation effects) was potentially misleading. We have removed Figure 4 from the manuscript, as it depicted non-significant interaction effects and did not add meaningful information. We have revised the Methods section to clarify that interactions were tested but not significant, and therefore simple slopes were not probed (Lines 460-461). |
| Similarly, Figure 2 seems to be not referenced in the text. The figures seem to show both significant and non-significant results; this approach to presenting the results should be indicated in the text. | We appreciate this feedback. To address this, we have added explicit references to all figures in the text (Lines 502-506; 539-544). We have also updated all figure captions to clearly indicate which paths/associations are significant versus non-significant. |
| Some of the recommendations for future research in the discussion section are analyses the authors could conduct with their dataset. This underscores the need to re-run the analyses or provide a stronger rationale for the model and measurement approach used in the manuscript. | We appreciate this comment. The future directions we propose require measures not available at the relevant time points in our dataset or alternative model specifications beyond the scope of the current hypotheses.  Our analytic rationale is provided in the Methods section, including: 1) justification for modeling EC as a latent construct using three subscale indicators to capture shared variance while accounting for measurement error, and 2) justification for testing mother and father effects separately based on formal dyadic equivalence testing via chi-square difference tests. We discuss model fit limitations and their implications for interpretation in the Discussion (Lines 659-666), noting that results should be interpreted cautiously and that alternative model specifications could reveal different associations. |
| **Reviewer #2:** This paper presents a large scale and impressive longitudinal study in which behavioral and biological data was collected at different time-points. It is well written and the scholarship is impressive. | We thank Reviewer 2 for the critical and rigorous evaluation of our manuscript. The concerns raised have led to substantial improvements in clarity, transparency, and appropriate caution in our interpretations. |
| In the correlation table (Table 1) variable 12 is BM cortisol slope. This is not presented in the description of measures; and seems unlikely because the other BM measures are very indirect. | Thank you for catching this inconsistency. We revised the Methods to ensure that all variables appearing in Table 1 are explicitly described, including birth mother cortisol slope where applicable (Lines 415-418). |
| In addition variables 1 and 2 are categorical -sex and ethnicity. I am not clear what meaning to attach to correlations between categorical and interval or continuous variables. | This is an excellent and important methodological point. We have clarified the types of correlation coefficients used for different variable types in both the Table 1 caption and the Methods section. Specifically, Pearson correlations are reported for continuous variables and that point-biserial correlations are reported for associations between categorical variables (child sex and race/ethnicity) and continuous variables, and we specify the coding for sex and the dummy coding approach for race/ethnicity (Lines 490-497). |
| The assertion that BM life stress, collected at various times post partum reflect genetic influences on the child's developing temperament is not clear. If this stress was affecting mothers when they were pregnant why assume the influence is genetic and not intrauterine? This assertion is in the title as well as argued throughout the paper, and is puzzling throughout. | We appreciate this important point and agree that adoption designs cannot fully disentangle genetic transmission from prenatal (intrauterine) environmental mechanisms when birth mother characteristics are used as predictors. To address this, we revised the conceptual framing throughout the manuscript to explicitly acknowledge that associations between birth mother characteristics and child outcomes may reflect multiple pathways. Throughout the Discussion and Conclusion, we consistently use language acknowledging both pathways. We also revised the title to explicitly distinguish "Heritable, Prenatal, and Environmental Pathways" to make this distinction clear from the outset. |
| The result that adoptive father's stress and not adoptive mother's stress at AC's 27 months of age moderates the child's EC at 11 makes no sense. The authors try to lay the groundwork for this counter-intuitive finding in the introduction but it remains unconvincing. It looks to the reader as if many different measures might have been modelled and that this finding is an artifact, a form of cherry picking. I wonder if this study was pre-registered. | We appreciate the reviewer's comment and have revised the manuscript to improve transparency and prevent misinterpretation. First, we want to clarify that adoptive father stress did **not** moderate the relationship between birth mother life stress and child EC. The moderation hypothesis was not supported for either adoptive mothers or adoptive fathers; all interaction terms were non-significant. What we did find was a direct effect of adoptive father stressful life events on child cortisol slope (β = 0.158, p = .030), but this was not part of a significant moderation or mediation pathway. We have substantially revised the Discussion to interpret this finding cautiously, explicitly acknowledging that: 1) the effect emerged from separate models for mothers and fathers rather than a direct statistical test comparing maternal versus paternal effects within a single model, 2) given the number of pathways tested and the modest effect size, we cannot entirely rule out Type I error, and 3) the overall model fit indices (CFI = .76) suggest the models do not fully capture the data structure, limiting confidence in specific parameter estimates (Lines 659-666). Nevertheless, we note that this finding demonstrates some consistency across analytic approaches (significant at both bivariate and multivariate levels, as shown in Table 1) and aligns with emerging evidence of father-specific influences on child stress physiology cited from prior research. We emphasize that this unexpected finding underscores the need for replication in independent samples and more direct comparisons of maternal versus paternal stress effects using models that simultaneously estimate both parent effects (Lines 667-674; 677-682). The study was not preregistered, and we have added this to the Limitations section. We clarified the rationale for examining mother and father effects separately in the Statistical Analysis section (Lines 745-752), noting that this approach was theory-informed and based on a formal test of dyadic equivalence via chi-square difference test (χ²Δ [1] = 3.9, p = .04) rather than post hoc selection (Lines 442-448). We believe these revisions address the concern while maintaining appropriate scientific caution. |
