# Supplementary material for: Heritable, Prenatal, and Environmental Pathways of Parental Stress: Differential Influences on Children’s Effortful Control and Cortisol Regulation
Source: PLoS One. 2026 Aug 11;21(8):e0355546. doi: 10.1371/journal.pone.0355546 (PMC13460627; doi:10.1371/journal.pone.0355546)
Supplement: Supplementary file 2 [file pone.0355546.s003.docx]

June 29, 2026

Ralph C. A. Rippe, Ph.D.

Academic Editor

PLOS One

Dear Dr. Rippe,

Thank you for the opportunity to revise and resubmit our manuscript titled, *Heritable, Prenatal, and Environmental Pathways of Parental Stress: Differential Influences on Children’s Effortful Control and Cortisol Regulation* (please note the updated title to accommodate one of the Reviewer 2’s points). We appreciate the thorough review and have aimed to address all the reviewer’s concerns. As a result, we believe the revised manuscript is significantly stronger. Our responses to Reviewer #2’s comments are detailed below (Reviewer #1 did not suggest any additional changes).

Sincerely,

Rebecca Gordon

Corresponding author

| Comments | Responses |
| --- | --- |
| **Reviewer #2:** I wrote in the original review that BM's cortisol slope was not presented in the measures section of the methods. When was it measured relative to the birth of the child? How many of the BMs participated in that part of the study? You wrote in your rebuttal that in lines 415-418 it is now fully presented and described, but it is not. | We sincerely thank Reviewer #2 for the careful read of our revision. We have now made substantial changes to address each point comprehensively.  We apologize for the incomplete description in our previous revision. We have now added a complete description of BM cortisol, including age of child at the time of measurement and total *N* (Lines 405-408). |
| The description of the rationale for the measures used is very well written, but the description of the measures themselves though improved is not. | We have revised the measure descriptions to ensure consistency, clarity, and completeness (Lines 292-306; 399-408). |
| There is still an overemphasis on genetics over intrauterine environment which is not theoretically or otherwise justified. | We have revised the theoretical premise underlying our rationale of predictors to acknowledge that the biologically transmitted influences from birth mothers’ stress experiences may arise from both genetic influences and prenatal pathways, such as developmental cues and hormones resulting from chronic dysregulation from previous or concurrent stress experiences.  We have clarified this issue in several relevant sections of the manuscript text (e.g., Lines 4, 21, 34-40, 50-54, 85-86, 142-144, 150, 154, 167, 222, 298-300, 560-565, 573-574, 576-580, 617, 662-664, 671, 679, 723, 726-729, 733). |
| You have a triple negative in the abstract which is unnecessary and confusing - line 16, I would lose the word "not" at the end. | We thank the reviewer for catching the triple negative. Accordingly, we have revised Line 16 to read: "Structural equation modeling revealed that birth mother life stress was linked to worse adoptee EC in adolescence; neither adoptive mothers’ nor fathers’ experiences of stressful life events predicted adoptee EC." |
