# Supplementary material for: Heritable, Prenatal, and Environmental Pathways of Parental Stress: Differential Influences on Children’s Effortful Control and Cortisol Regulation
Source: PLoS One. 2026 Aug 11;21(8):e0355546. doi: 10.1371/journal.pone.0355546 (PMC13460627; doi:10.1371/journal.pone.0355546)
Supplement: Peer review history

# Peer review: Decision Letter 0

**DOI:** 10.1371/journal.pone.0355546.r001

**Authors:** Ralph C. A. Rippe

25 Mar 2026

PONE-D-25-57651

Heritable and Environmental Pathways of Parental Stress: Differential Influences on Children’s Effortful Control and Cortisol Regulation

PLOS One

Dear Dr. Gordon,

Thank you for submitting your manuscript to PLOS ONE. After careful consideration, we feel that it has merit but does not fully meet PLOS ONE’s publication criteria as it currently stands.

I have read your work, and two independent experts have also shared their opinion.

Although the reviewers report positively on the potential of the current design, they also agree in their observations that many aspects of the description of measures and design choices are reported in a unclear, incomplete or even contradictory manner. I concur with the suggestions for explicitation of design choices and, among other things, more exact description of statistical measures (e.g. the example from reviewer 2 on different correlation coefficients), clarification and deeper argumentation on the disentanglement of genetic and environmental influences. Both reviewers brought this up using different wordings and angles.

If you feel that you can address the comments, I would like to invite you to submit a major revision of the manuscript accordingly, with an emphasis on overall clarification and alignment of the theoretical background, the possibilities of the longitudinal design and the final conclusions.

Please submit your revised manuscript by May 09 2026 11:59PM. If you will need more time than this to complete your revisions, please reply to this message or contact the journal office at plosone@plos.org. When you're ready to submit your revision, log on to https://www.editorialmanager.com/pone/ and select the 'Submissions Needing Revision' folder to locate your manuscript file.

Please include the following items when submitting your revised manuscript:

- A letter that responds to each point raised by the academic editor and reviewer(s). You should upload this letter as a separate file labeled 'Response to Reviewers'.
- A marked-up copy of your manuscript that highlights changes made to the original version. You should upload this as a separate file labeled 'Revised Manuscript with Track Changes'.
- An unmarked version of your revised paper without tracked changes. You should upload this as a separate file labeled 'Manuscript'.

If you would like to make changes to your financial disclosure, please include your updated statement in your cover letter. Guidelines for resubmitting your figure files are available below the reviewer comments at the end of this letter.

If applicable, we recommend that you deposit your laboratory protocols in protocols.io to enhance the reproducibility of your results. Protocols.io assigns your protocol its own identifier (DOI) so that it can be cited independently in the future. For instructions see: https://journals.plos.org/plosone/s/submission-guidelines#loc-laboratory-protocols. Additionally, PLOS ONE offers an option for publishing peer-reviewed Lab Protocol articles, which describe protocols hosted on protocols.io. Read more information on sharing protocols at https://plos.org/protocols?utm_medium=editorial-email&utm_source=authorletters&utm_campaign=protocols.

As the corresponding author, your ORCID iD is verified in the submission system and will appear in the published article. PLOS supports the use of ORCID, and we encourage all coauthors to register for an ORCID iD and use it as well. Please encourage your coauthors to verify their ORCID iD within the submission system before final acceptance, as unverified ORCID iDs will not appear in the published article. *Only*  the individual author can complete the verification step; PLOS staff *cannot*  verify ORCID iDs on behalf of authors.

We look forward to receiving your revised manuscript.

Kind regards,

Ralph C. A. Rippe, Ph.D.

Academic Editor

PLOS One

Journal Requirements:

When submitting your revision, we need you to address these additional requirements.

1. Please ensure that your manuscript meets PLOS ONE's style requirements, including those for file naming. The PLOS ONE style templates can be found at

https://journals.plos.org/plosone/s/file?id=wjVg/PLOSOne_formatting_sample_main_body.pdf and

https://journals.plos.org/plosone/s/file?id=ba62/PLOSOne_formatting_sample_title_authors_affiliations.pdf

2. Thank you for stating the following financial disclosure: [Data collection for this study were supported by grant R56 HD042608 and R01 HD042608 from the Eunice Kennedy Shriver National Institute of Child Health & Human Development, the National Institute on Drug Abuse, and OBSSR, NIH, U.S. PHS (PI: Leslie Leve, PhD); by grant R01 DA035062 from the National Institute on Drug Abuse (PI: Leslie Leve, PhD) and grant R01 DA020585 from the National Institute on Drug Abuse, the National Institute of Mental Health, and OBSSR, NIH, U.S. PHS (PI: Jenae Neiderhiser, PhD); and by the Environmental influences on Child Health Outcomes program, Office of The Director, National Institutes of Health, under Award Number UH3OD023389 (PIs: Leslie Leve, PhD; Jenae Neiderhiser, PhD; Jody Ganiban, PhD).].

Please state what role the funders took in the study.  If the funders had no role, please state: ""The funders had no role in study design, data collection and analysis, decision to publish, or preparation of the manuscript.""

If this statement is not correct you must amend it as needed.

Please include this amended Role of Funder statement in your cover letter; we will change the online submission form on your behalf.

3. In the online submission form you indicate that your data is not available for proprietary reasons and have provided a contact point for accessing this data. Please note that your current contact point is a co-author on this manuscript. According to our Data Policy, the contact point must not be an author on the manuscript and must be an institutional contact, ideally not an individual. Please revise your data statement to a non-author institutional point of contact, such as a data access or ethics committee, and send this to us via return email. Please also include contact information for the third party organization, and please include the full citation of where the data can be found.

4. When completing the data availability statement of the submission form, you indicated that you will make your data available on acceptance. We strongly recommend all authors decide on a data sharing plan before acceptance, as the process can be lengthy and hold up publication timelines. Please note that, though access restrictions are acceptable now, your entire data will need to be made freely accessible if your manuscript is accepted for publication. This policy applies to all data except where public deposition would breach compliance with the protocol approved by your research ethics board. If you are unable to adhere to our open data policy, please kindly revise your statement to explain your reasoning and we will seek the editor's input on an exemption. Please be assured that, once you have provided your new statement, the assessment of your exemption will not hold up the peer review process.

5. Please amend either the abstract on the online submission form (via Edit Submission) or the abstract in the manuscript so that they are identical.

6. Please include your full ethics statement in the ‘Methods’ section of your manuscript file. In your statement, please include the full name of the IRB or ethics committee who approved or waived your study, as well as whether or not you obtained informed written or verbal consent. If consent was waived for your study, please include this information in your statement as well.

7. If the reviewer comments include a recommendation to cite specific previously published works, please review and evaluate these publications to determine whether they are relevant and should be cited. There is no requirement to cite these works unless the editor has indicated otherwise.

[Note: HTML markup is below. Please do not edit.]

Reviewers' comments:

Reviewer's Responses to Questions

**Comments to the Author**

1. Is the manuscript technically sound, and do the data support the conclusions?

The manuscript must describe a technically sound piece of scientific research with data that supports the conclusions. Experiments must have been conducted rigorously, with appropriate controls, replication, and sample sizes. The conclusions must be drawn appropriately based on the data presented.

Reviewer #1: Partly

Reviewer #2: No

**********

2. Has the statistical analysis been performed appropriately and rigorously?

Reviewer #1: Yes

Reviewer #2: No

**********

3. Have the authors made all data underlying the findings in their manuscript fully available?

The PLOS Data policy requires authors to make all data underlying the findings described in their manuscript fully available without restriction, with rare exception (please refer to the Data Availability Statement in the manuscript PDF file). The data should be provided as part of the manuscript or its supporting information, or deposited to a public repository. For example, in addition to summary statistics, the data points behind means, medians and variance measures should be available. If there are restrictions on publicly sharing data—e.g. participant privacy or use of data from a third party—those must be specified.

Reviewer #1: No

Reviewer #2: Yes

**********

4. Is the manuscript presented in an intelligible fashion and written in standard English?

PLOS ONE does not copyedit accepted manuscripts, so the language in submitted articles must be clear, correct, and unambiguous. Any typographical or grammatical errors should be corrected at revision, so please note any specific errors here.

Reviewer #1: Yes

Reviewer #2: Yes

**********

5. Review Comments to the Author

Please use the space provided to explain your answers to the questions above. You may also include additional comments for the author, including concerns about dual publication, research ethics, or publication ethics. (Please upload your review as an attachment if it exceeds 20,000 characters)

Reviewer #1: This manuscript is well written and the study has many strengths including a large sample size and use of a longitudinal behavioral genetics study design (parent-offspring adoption design).

The purpose of including adoptive mothers/fathers report of stressful life experiences should be clarified in the manuscript. This construct was measured with one questionnaire whereas other main variables in the model were measured using latent variables with multiple indicators. This approach seems to detract from the conclusions that can be made from analyses of parental stressful life events, especially when compared to the more robust measurement approach used for the other variables. This is also problematic as part of the aims of the study center on genetic vs. environmental influences.

The introduction should differentiate between positive and toxic stress such as manageable vs. overwhelming levels of stress rather than defining all stress as harmful.

It would be helpful to explain the focus on effortful control earlier in the manuscript. In addition, in multiple places, it seems as though studying adolescent mental health (rather than effortful control) could be more aligned with the authors’ interests and hypotheses.

On Line 90 of the introduction, it would be helpful to clarify what is meant by “…which peak during adolescence.” It would help to explain more about the development of effortful control before, during, and after adolescence. In that same section, the difference between effortful control and executive function should be explained in more detail. The two constructs are overlapping, and some argue that they are different terms for the same underlying construct.

It is important to include in the introduction that studies of salivary cortisol metrics such as diurnal cortisol slope have yielded inconsistent results.

Is there data on effortful control prior to adolescence? Including those data could strengthen the model.

What is shown in Figure 4 is not clear. It would help to clarify the reason for including this figure. Similarly, Figure 2 seems to be not referenced in the text. The figures seem to show both significant and non-significant results; this approach to presenting the results should be indicated in the text.

Some of the recommendations for future research in the discussion section are analyses the authors could conduct with their dataset. This underscores the need to re-run the analyses or provide a stronger rationale for the model and measurement approach used in the manuscript.

Reviewer #2: This paper presents a large scale and impressive longitudinal study in which behavioral and biological data was collected at different time-points.

It is well written and the scholarship is impressive.

However I have major reservations about this manuscript:

1. In the correlation table (Table 1) variable 12 is BM cortisol slope. This is not presented in the description of measures; and seems unlikely because the other BM measures are very indirect. In addition variables 1 and 2 are categorical -sex and ethnicity. I am not clear what meaning to attach to correlations between categorical and interval or continuous variables.

2. The assertion that BM life stress, collected at various times post partum reflect genetic influences on the child's developing temperament is not clear. If this stress was affecting mothers when they were pregnant why assume the influence is genetic and not intrauterine? This assertion is in the title as well as argued throughout the paper, and is puzzling throughout.

3. The result that adoptive father's stress and not adoptive mother's stress at AC's 27 months of age moderates the child's EC at 11 makes no sense. The authors try to lay the groundwork for this counter-intuitive finding in the introduction but it remains unconvincing. It looks to the reader as if many different measures might have been modelled and that this finding is an artifact, a form of cherry picking. I wonder if this study was pre-registered.

In summary, this paper seems to me to not do justice to the scope of the longitudinal study it reports on, and to make unfounded assertions.

**********

6. PLOS authors have the option to publish the peer review history of their article (what does this mean?). If published, this will include your full peer review and any attached files.

If you choose “no”, your identity will remain anonymous but your review may still be made public.

**Do you want your identity to be public for this peer review?**  For information about this choice, including consent withdrawal, please see our Privacy Policy.

Reviewer #1: No

Reviewer #2: No

**********

[NOTE: If reviewer comments were submitted as an attachment file, they will be attached to this email and accessible via the submission site. Please log into your account, locate the manuscript record, and check for the action link "View Attachments". If this link does not appear, there are no attachment files.]

To ensure your figures meet our technical requirements, please review our figure guidelines: https://journals.plos.org/plosone/s/figures

You may also use PLOS’s free figure tool, NAAS, to help you prepare publication quality figures: https://journals.plos.org/plosone/s/figures#loc-tools-for-figure-preparation.

NAAS will assess whether your figures meet our technical requirements by comparing each figure against our figure specifications.

# Author comment: Author response to Decision Letter 1

**DOI:** 10.1371/journal.pone.0355546.r002

5 May 2026

Thank you for the opportunity to revise and resubmit our manuscript titled, Heritable, Prenatal, and Environmental Pathways of Parental Stress: Differential Influences on Children’s Effortful Control and Cortisol Regulation. We appreciate the thorough review and feedback we received. We have done our best to address all the reviewers’ concerns and believe the resulting manuscript is significantly strengthened. Our responses to reviewer comments are detailed below.

Journal Requirements: Please ensure that your manuscript meets PLOS ONE's style requirements, including those for file naming.

Response: We have reviewed and ensured that all file naming and formatting complies with PLOS ONE style requirements.

Thank you for stating the following financial disclosure: [Data collection for this study were supported by grant R56 HD042608 and R01 HD042608 from the Eunice Kennedy Shriver National Institute of Child Health & Human Development, the National Institute on Drug Abuse, and OBSSR, NIH, U.S. PHS (PI: Leslie Leve, PhD); by grant R01 DA035062 from the National Institute on Drug Abuse (PI: Leslie Leve, PhD) and grant R01 DA020585 from the National Institute on Drug Abuse, the National Institute of Mental Health, and OBSSR, NIH, U.S. PHS (PI: Jenae Neiderhiser, PhD); and by the Environmental influences on Child Health Outcomes program, Office of The Director, National Institutes of Health, under Award Number UH3OD023389 (PIs: Leslie Leve, PhD; Jenae Neiderhiser, PhD; Jody Ganiban, PhD).].

Please state what role the funders took in the study. If the funders had no role, please state: ""The funders had no role in study design, data collection and analysis, decision to publish, or preparation of the manuscript.""

If this statement is not correct you must amend it as needed.

Please include this amended Role of Funder statement in your cover letter; we will change the online submission form on your behalf.

Response: We have added the following statement to the cover letter and will update the online submission form accordingly: "The funders had no role in study design, data collection and analysis, decision to publish, or preparation of the manuscript."

In the online submission form you indicate that your data is not available for proprietary reasons and have provided a contact point for accessing this data. Please note that your current contact point is a co-author on this manuscript. According to our Data Policy, the contact point must not be an author on the manuscript and must be an institutional contact, ideally not an individual. Please revise your data statement to a non-author institutional point of contact, such as a data access or ethics committee, and send this to us via return email. Please also include contact information for the third party organization, and please include the full citation of where the data can be found.

Response: We have modified this in order to post a public dataset. Specifically, the de-identified dataset and analysis code are now publicly available via OSF (https://osf.io/usw93).

When completing the data availability statement of the submission form, you indicated that you will make your data available on acceptance. We strongly recommend all authors decide on a data sharing plan before acceptance, as the process can be lengthy and hold up publication timelines. Please note that, though access restrictions are acceptable now, your entire data will need to be made freely accessible if your manuscript is accepted for publication. This policy applies to all data except where public deposition would breach compliance with the protocol approved by your research ethics board. If you are unable to adhere to our open data policy, please kindly revise your statement to explain your reasoning and we will seek the editor's input on an exemption. Please be assured that, once you have provided your new statement, the assessment of your exemption will not hold up the peer review process.

Response: We confirm our commitment to PLOS ONE's open data policy. As stated above, the de-identified dataset and analysis code are now publicly available via OSF (https://osf.io/usw93). We have consulted with our IRB and confirmed that public sharing of de-identified data is consistent with our approved protocol (University of Oregon IRB #08082016.007) and participant consent procedures.

Please include your full ethics statement in the ‘Methods’ section of your manuscript file. In your statement, please include the full name of the IRB or ethics committee who approved or waived your study, as well as whether or not you obtained informed written or verbal consent. If consent was waived for your study, please include this information in your statement as well.

Response: The ethics statement is now included in the Methods section and specifies the approving IRB and the consent procedure (Lines 289-298)

Reviewer #1:

This manuscript is well written and the study has many strengths including a large sample size and use of a longitudinal behavioral genetics study design (parent-offspring adoption design).

Response: We thank Reviewer 1 for the constructive feedback and specific suggestions for improving clarity and comprehensiveness.

The purpose of including adoptive mothers/fathers report of stressful life experiences should be clarified in the manuscript. This construct was measured with one questionnaire whereas other main variables in the model were measured using latent variables with multiple indicators. This approach seems to detract from the conclusions that can be made from analyses of parental stressful life events, especially when compared to the more robust measurement approach used for the other variables. This is also problematic as part of the aims of the study center on genetic vs. environmental influences.

Response: We appreciate the reviewer’s point and have clarified the purpose of adoptive parent stressful life experiences in the Introduction (Lines 141-147; 162-164; 228-230). This variable is critical to disentangle the heritable/prenatal (birth parent) effects from the postnatal rearing environmental (adoptive parent) stress effects. We specifically selected discrete stressful life events as our environmental stress indicator because life events represent objectively verifiable environmental exposures that are conceptually distinct from parenting behaviors, parent-child relationship quality, or heritable child characteristics. In order to characterize variation in adoptive parent stressful life experiences, we were able to capitalize on the available measures of adoptive parent stressful life experiences in EGDS during the relevant developmental window (27 months). While multi-indicator latent constructs may provide more robust measurement, modeling this variable as a single indicator was the appropriate approach given the available data.

The introduction should differentiate between positive and toxic stress such as manageable vs. overwhelming levels of stress rather than defining all stress as harmful.

Response: We appreciate the reviewer's point. Our Introduction references differential susceptibility theory, which does acknowledge that some children may benefit from positive environments. However, the present study focuses specifically on adverse stress exposure rather than examining the full continuum of environmental quality. Our measures (ACEs, negative life events, material hardship) are validated risk indices designed to capture experiences such as abuse, neglect, and financial instability that have been consistently linked to poor developmental outcomes. These are not measures of normative challenges or positive experiences. While differential susceptibility theory suggests that prenatal stress may heighten sensitivity to both adverse and supportive contexts, testing the "for better" hypothesis would require measures of positive caregiving experiences (e.g., warmth, sensitivity, enrichment), which were not the focus of the present analyses. To clarify our scope, we revised the opening paragraph to specify "chronic or severe stress exposure" (Line 31).

It would be helpful to explain the focus on effortful control earlier in the manuscript.

Response: We appreciate this feedback. Our Introduction includes a dedicated section titled "The Role of Effortful Control in Adolescence" that provides a comprehensive definition of EC, its components (inhibitory control and attention regulation), and its theoretical importance before presenting our study hypotheses. This organizational structure allows us to first establish the broader conceptual framework (stress transmission pathways, HPA axis functioning, and adoption designs for disentangling heritable from environmental influences) before focusing on the specific self-regulation outcome (EC) being examined. We believe this structure provides readers with the necessary theoretical context to understand why EC is an appropriate outcome for examining the effects of intergenerational stress transmission.

In addition, in multiple places, it seems as though studying adolescent mental health (rather than effortful control) could be more aligned with the authors’ interests and hypotheses.

Response: We appreciate the reviewer's perspective. However, effortful control (EC) was selected as the primary outcome based on strong theoretical and empirical foundations. While mental health outcomes are certainly important, EC represents a more proximal and mechanistic target that can inform our understanding of how parental stress shapes children’s self-regulatory capacities. We have clarified the rationale for focusing on EC in the Introduction (Lines 39-42).

On Line 90 of the introduction, it would be helpful to clarify what is meant by “…which peak during adolescence.” It would help to explain more about the development of effortful control before, during, and after adolescence.

Response: We have added a developmental trajectory section to clarify EC development across the lifespan (Lines 108-112).

In that same section, the difference between effortful control and executive function should be explained in more detail. The two constructs are overlapping, and some argue that they are different terms for the same underlying construct.

Response: We agree and have expanded the conceptual framing to distinguish EC and executive function while acknowledging their substantial overlap (Lines 98-105).

It is important to include in the introduction that studies of salivary cortisol metrics such as diurnal cortisol slope have yielded inconsistent results.

Response: Excellent point. We have added acknowledgment of inconsistent cortisol findings in the HPA Axis section (Lines 191-198).

Is there data on effortful control prior to adolescence? Including those data could strengthen the model.

Response: We appreciate this suggestion. The current study was designed to examine adolescent EC as the outcome of interest, specifically focusing on how early stress exposures (birth mother life stress and adoptive parent stressful life events during toddlerhood) predict self-regulatory capacity in adolescence. We focused on age 11 because EC skills, including inhibitory control and attention regulation, peak during adolescence (as mentioned in the Introduction), making this developmental period ideal for examining the cumulative effects of early stress exposure on self-regulatory outcomes. Examining developmental trajectories of EC across childhood would be a valuable direction for future research but is beyond the scope of the current study's research questions, which focus on adolescent regulatory outcomes as a function of early heritable and environmental stress exposure.

What is shown in Figure 4 is not clear. It would help to clarify the reason for including this figure.

Response: We agree that Figure 4 (loop plot of non-significant moderation effects) was potentially misleading. We have removed Figure 4 from the manuscript, as it depicted non-significant interaction effects and did not add meaningful information. We have revised the Methods section to clarify that interactions were tested but not significant, and therefore simple slopes were not probed (Lines 460-461).

Similarly, Figure 2 seems to be not referenced in the text. The figures seem to show both significant and non-significant results; this approach to presenting the results should be indicated in the text.

Response: We appreciate this feedback. To address this, we have added explicit references to all figures in the text (Lines 502-506; 539-544). We have also updated all figure captions to clearly indicate which paths/associations are significant versus non-significant.

Some of the recommendations for future research in the discussion section are analyses the authors could conduct with their dataset. This underscores the need to re-run the analyses or provide a stronger rationale for the model and measurement approach used in the manuscript.

Response: We appreciate this comment. The future directions we propose require measures not available at the relevant time points in our dataset or alternative model specifications beyond the scope of the current hypotheses.

Our analytic rationale is provided in the Methods section, including: 1) justification for modeling EC as a latent construct using three subscale indicators to capture shared variance while accounting for measurement error, and 2) justification for testing mother and father effects separately based on formal dyadic equivalence testing via chi-square difference tests. We discuss model fit limitations and their implications for interpretation in the Discussion (Lines 659-666), noting that results should be interpreted cautiously and that alternative model specifications could reveal different associations.

Reviewer #2:

This paper presents a large scale and impressive longitudinal study in which behavioral and biological data was collected at different time-points. It is well written and the scholarship is impressive.

Response: We thank Reviewer 2 for the critical and rigorous evaluation of our manuscript. The concerns raised have led to substantial improvements in clarity, transparency, and appropriate caution in our interpretations.

In the correlation table (Table 1) variable 12 is BM cortisol slope. This is not presented in the description of measures; and seems unlikely because the other BM measures are very indirect.

Response: Thank you for catching this inconsistency. We revised the Methods to ensure that all variables appearing in Table 1 are explicitly described, including birth mother cortisol slope where applicable (Lines 415-418).

In addition variables 1 and 2 are categorical -sex and ethnicity. I am not clear what meaning to attach to correlations between categorical and interval or continuous variables.

Response: This is an excellent and important methodological point. We have clarified the types of correlation coefficients used for different variable types in both the Table 1 caption and the Methods section. Specifically, Pearson correlations are reported for continuous variables and that point-biserial correlations are reported for associations between categorical variables (child sex and race/ethnicity) and continuous variables, and we specify the coding for sex and the dummy coding approach for race/ethnicity (Lines 490-497).

The assertion that BM life stress, collected at various times post partum reflect genetic influences on the child's developing temperament is not clear. If this stress was affecting mothers when they were pregnant why assume the influence is genetic and not intrauterine? This assertion is in the title as well as argued throughout the paper, and is puzzling throughout.

Response: We appreciate this important point and agree that adoption designs cannot fully disentangle genetic transmission from prenatal (intrauterine) environmental mechanisms when birth mother characteristics are used as predictors. To address this, we revised the conceptual framing throughout the manuscript to explicitly acknowledge that associations between birth mother characteristics and child outcomes may reflect multiple pathways. Throughout the Disc

Attachment Submitted filename: PLOS ONE Peer Review Response.docx

# Peer review: Decision Letter 1

**DOI:** 10.1371/journal.pone.0355546.r003

**Authors:** Ralph C. A. Rippe

31 May 2026

PONE-D-25-57651R1

Heritable, Prenatal, and Environmental Pathways of Parental Stress: Differential Influences on Children’s Effortful Control and Cortisol Regulation

PLOS One

Dear Dr. Gordon,

Thank you for submitting your manuscript to PLOS ONE. After careful consideration, we feel that it has merit but since not all reviewer comments have been addressed (while announced in the rebuttal letter), the manuscript currently does not fully meet PLOS ONE’s publication criteria.  Therefore, we invite you to submit a revised version of the manuscript that addresses the points raised by reviewer 2 during the review process.

Please submit your revised manuscript by Jul 15 2026 11:59PM. If you will need more time than this to complete your revisions, please reply to this message or contact the journal office at plosone@plos.org. When you're ready to submit your revision, log on to https://www.editorialmanager.com/pone/ and select the 'Submissions Needing Revision' folder to locate your manuscript file.

Please include the following items when submitting your revised manuscript:

- A letter that responds to each point raised by the academic editor and reviewer(s). You should upload this letter as a separate file labeled 'Response to Reviewers'.
- A marked-up copy of your manuscript that highlights changes made to the original version. You should upload this as a separate file labeled 'Revised Manuscript with Track Changes'.
- An unmarked version of your revised paper without tracked changes. You should upload this as a separate file labeled 'Manuscript'.

If you would like to make changes to your financial disclosure, please include your updated statement in your cover letter. Guidelines for resubmitting your figure files are available below the reviewer comments at the end of this letter.

If applicable, we recommend that you deposit your laboratory protocols in protocols.io to enhance the reproducibility of your results. Protocols.io assigns your protocol its own identifier (DOI) so that it can be cited independently in the future. For instructions see: https://journals.plos.org/plosone/s/submission-guidelines#loc-laboratory-protocols. Additionally, PLOS ONE offers an option for publishing peer-reviewed Lab Protocol articles, which describe protocols hosted on protocols.io. Read more information on sharing protocols at https://plos.org/protocols?utm_medium=editorial-email&utm_source=authorletters&utm_campaign=protocols.

As the corresponding author, your ORCID iD is verified in the submission system and will appear in the published article. PLOS supports the use of ORCID, and we encourage all coauthors to register for an ORCID iD and use it as well. Please encourage your coauthors to verify their ORCID iD within the submission system before final acceptance, as unverified ORCID iDs will not appear in the published article. *Only*  the individual author can complete the verification step; PLOS staff *cannot*  verify ORCID iDs on behalf of authors.

We look forward to receiving your revised manuscript.

Kind regards,

Ralph C. A. Rippe, Ph.D.

Academic Editor

PLOS One

Journal Requirements:

If the reviewer comments include a recommendation to cite specific previously published works, please review and evaluate these publications to determine whether they are relevant and should be cited. There is no requirement to cite these works unless the editor has indicated otherwise.

[Note: HTML markup is below. Please do not edit.]

Reviewers' comments:

Reviewer's Responses to Questions

**Comments to the Author**

1. If the authors have adequately addressed your comments raised in a previous round of review and you feel that this manuscript is now acceptable for publication, you may indicate that here to bypass the “Comments to the Author” section, enter your conflict of interest statement in the “Confidential to Editor” section, and submit your "Accept" recommendation.

Reviewer #1: All comments have been addressed

Reviewer #2: (No Response)

**********

2. Is the manuscript technically sound, and do the data support the conclusions?

The manuscript must describe a technically sound piece of scientific research with data that supports the conclusions. Experiments must have been conducted rigorously, with appropriate controls, replication, and sample sizes. The conclusions must be drawn appropriately based on the data presented.

Reviewer #1: Yes

Reviewer #2: Partly

**********

3. Has the statistical analysis been performed appropriately and rigorously?

Reviewer #1: Yes

Reviewer #2: I Don't Know

**********

4. Have the authors made all data underlying the findings in their manuscript fully available?

The PLOS Data policy requires authors to make all data underlying the findings described in their manuscript fully available without restriction, with rare exception (please refer to the Data Availability Statement in the manuscript PDF file). The data should be provided as part of the manuscript or its supporting information, or deposited to a public repository. For example, in addition to summary statistics, the data points behind means, medians and variance measures should be available. If there are restrictions on publicly sharing data—e.g. participant privacy or use of data from a third party—those must be specified.

Reviewer #1: Yes

Reviewer #2: Yes

**********

5. Is the manuscript presented in an intelligible fashion and written in standard English?

PLOS ONE does not copyedit accepted manuscripts, so the language in submitted articles must be clear, correct, and unambiguous. Any typographical or grammatical errors should be corrected at revision, so please note any specific errors here.

Reviewer #1: Yes

Reviewer #2: Yes

**********

6. Review Comments to the Author

Please use the space provided to explain your answers to the questions above. You may also include additional comments for the author, including concerns about dual publication, research ethics, or publication ethics. (Please upload your review as an attachment if it exceeds 20,000 characters)

Reviewer #1: (No Response)

Reviewer #2: Some of my concerns but not all have been answered. Here is an example

I wrote in the original review that BM's cortisol slope was not presented in the measures section of the methods. When was it measured relative to the birth of the child? How many of the BMs participated in that part of the study? You wrote in your rebuttal that in lines 415-418 it is now fully presented and described, but it is not.

The description of the rationale for the measures used is very well written, but the description of the measures themselves though improved is not.

There is still an overemphasis on genetics over intrauterine environment which is not theoretically or otherwise justified.

While the data is derived from a very impressive study, the current re-analyses are not impressive or convincing.

and a minor point - you have a triple negative in the abstract which is unnecessary and confusing - line 16 I would lose the word "not" at the end.

**********

7. PLOS authors have the option to publish the peer review history of their article (what does this mean?). If published, this will include your full peer review and any attached files.

If you choose “no”, your identity will remain anonymous but your review may still be made public.

**Do you want your identity to be public for this peer review?**  For information about this choice, including consent withdrawal, please see our Privacy Policy.

Reviewer #1: No

Reviewer #2: No

**********

[NOTE: If reviewer comments were submitted as an attachment file, they will be attached to this email and accessible via the submission site. Please log into your account, locate the manuscript record, and check for the action link "View Attachments". If this link does not appear, there are no attachment files.]

To ensure your figures meet our technical requirements, please review our figure guidelines: https://journals.plos.org/plosone/s/figures

You may also use PLOS’s free figure tool, NAAS, to help you prepare publication quality figures: https://journals.plos.org/plosone/s/figures#loc-tools-for-figure-preparation.

NAAS will assess whether your figures meet our technical requirements by comparing each figure against our figure specifications.

# Author comment: Author response to Decision Letter 2

**DOI:** 10.1371/journal.pone.0355546.r004

3 Jul 2026

Thank you for the opportunity to revise and resubmit our manuscript titled, Heritable, Prenatal, and Environmental Pathways of Parental Stress: Differential Influences on Children’s Effortful Control and Cortisol Regulation (please note the updated title to accommodate one of the Reviewer 2’s points). We appreciate the thorough review and have aimed to address all the reviewer’s concerns. As a result, we believe the revised manuscript is significantly stronger. Our responses to Reviewer #2’s comments are detailed below (Reviewer #1 did not suggest any additional changes).

Reviewer #2: I wrote in the original review that BM's cortisol slope was not presented in the measures section of the methods. When was it measured relative to the birth of the child? How many of the BMs participated in that part of the study? You wrote in your rebuttal that in lines 415-418 it is now fully presented and described, but it is not.

Response: We sincerely thank Reviewer #2 for the careful read of our revision. We have now made substantial changes to address each point comprehensively.

We apologize for the incomplete description in our previous revision. We have now added a complete description of BM cortisol, including age of child at the time of measurement and total N (Lines 405-408).

Reviewer #2: The description of the rationale for the measures used is very well written, but the description of the measures themselves though improved is not.

Response: We have revised the measure descriptions to ensure consistency, clarity, and completeness (Lines 292-306; 399-408).

Reviewer #2: There is still an overemphasis on genetics over intrauterine environment which is not theoretically or otherwise justified.

Response:We have revised the theoretical premise underlying our rationale of predictors to acknowledge that the biologically transmitted influences from birth mothers’ stress experiences may arise from both genetic influences and prenatal pathways, such as developmental cues and hormones resulting from chronic dysregulation from previous or concurrent stress experiences.

We have clarified this issue in several relevant sections of the manuscript text (e.g., Lines 4, 21, 34-40, 50-54, 85-86, 142-144, 150, 154, 167, 222, 298-300, 560-565, 573-574, 576-580, 617, 662-664, 671, 679, 723, 726-729, 733).

Reviewer #2: You have a triple negative in the abstract which is unnecessary and confusing - line 16, I would lose the word "not" at the end.

Response: We thank the reviewer for catching the triple negative. Accordingly, we have revised Line 16 to read: "Structural equation modeling revealed that birth mother life stress was linked to worse adoptee EC in adolescence; neither adoptive mothers’ nor fathers’ experiences of stressful life events predicted adoptee EC."

Attachment Submitted filename: PLOS ONE Peer Review Response_final.docx

# Editor report: Decision Letter 2

**DOI:** 10.1371/journal.pone.0355546.r005

**Authors:** Ralph C. A. Rippe

23 Jul 2026

Heritable, Prenatal, and Environmental Pathways of Parental Stress: Differential Influences on Children’s Effortful Control and Cortisol Regulation

PONE-D-25-57651R2

Dear Dr. Gordon,

Thank you for your strong efforts in response to the comments of Reviewer 2. For evaluation, reviewer 1 had already accepted after the previous revision, while reviewer 2 suggested a major revision. Both reviewers are currently no longer available for full revision of your second revision. However, after my own reading and looking back at all previous communication, I feel you have adequately addressed all requests by reviewer 2.

We’re pleased to inform you that your manuscript has been judged scientifically suitable for publication and will be formally accepted for publication once it meets all outstanding technical requirements.

Within one week, you’ll receive an e-mail detailing the required amendments. When these have been addressed, you’ll receive a formal acceptance letter and your manuscript will be scheduled for publication.

An invoice will be generated when your article is formally accepted. Please note, if your institution has a publishing partnership with PLOS and your article meets the relevant criteria, all or part of your publication costs will be covered. Please make sure your user information is up-to-date by logging into Editorial Manager at Editorial Manager® and clicking the ‘Update My Information' link at the top of the page. For questions related to billing, please contact billing support.

If your institution or institutions have a press office, please notify them about your upcoming paper to help maximize its impact. If they’ll be preparing press materials, please inform our press team as soon as possible -- no later than 48 hours after receiving the formal acceptance. Your manuscript will remain under strict press embargo until 2 pm Eastern Time on the date of publication. For more information, please contact onepress@plos.org.

Kind regards,

Ralph C. A. Rippe, Ph.D.

Academic Editor

PLOS One

# Editor report: Acceptance letter

**DOI:** 10.1371/journal.pone.0355546.r006

**Authors:** Ralph C. A. Rippe

PONE-D-25-57651R2

PLOS One

Dear Dr. Gordon,

I'm pleased to inform you that your manuscript has been deemed suitable for publication in PLOS One. Congratulations! Your manuscript is now being handed over to our production team.

At this stage, our production department will prepare your paper for publication. This includes ensuring the following:

* All references, tables, and figures are properly cited

* All relevant supporting information is included in the manuscript submission,

* There are no issues that prevent the paper from being properly typeset

You will receive further instructions from the production team, including instructions on how to review your proof when it is ready. Please keep in mind that we are working through a large volume of accepted articles, so please give us a few days to review your paper and let you know the next and final steps.

Lastly, if your institution or institutions have a press office, please let them know about your upcoming paper now to help maximize its impact. If they'll be preparing press materials, please inform our press team within the next 48 hours. Your manuscript will remain under strict press embargo until 2 pm Eastern Time on the date of publication. For more information, please contact onepress@plos.org.

You will receive an invoice from PLOS for your publication fee after your manuscript has reached the completed accept phase. If you receive an email requesting payment before acceptance or for any other service, this may be a phishing scheme. Learn how to identify phishing emails and protect your accounts at https://explore.plos.org/phishing.

If we can help with anything else, please email us at customercare@plos.org.

Thank you for submitting your work to PLOS ONE and supporting open access.

Kind regards,

PLOS ONE Editorial Office Staff

on behalf of

Dr. Ralph C. A. Rippe

Academic Editor

PLOS One
